# Dengue Virus Ensures Its Fusion in Late Endosomes Using Compartment-Specific Lipids

**DOI:** 10.1371/journal.ppat.1001131

**Published:** 2010-10-07

**Authors:** Elena Zaitseva, Sung-Tae Yang, Kamran Melikov, Sergei Pourmal, Leonid V. Chernomordik

**Affiliations:** Section on Membrane Biology, Laboratory of Cellular and Molecular Biophysics, Eunice Kennedy Shriver National Institute of Child Health and Human Development, National Institutes of Health, Bethesda, Maryland, United States of America; Washington University School of Medicine, United States of America

## Abstract

Many enveloped viruses invade cells via endocytosis and use different environmental factors as triggers for virus-endosome fusion that delivers viral genome into cytosol. Intriguingly, dengue virus (DEN), the most prevalent mosquito-borne virus that infects up to 100 million people each year, fuses only in late endosomes, while activation of DEN protein fusogen glycoprotein E is triggered already at pH characteristic for early endosomes. Are there any cofactors that time DEN fusion to virion entry into late endosomes? Here we show that DEN utilizes bis(monoacylglycero)phosphate, a lipid specific to late endosomes, as a co-factor for its endosomal acidification-dependent fusion machinery. Effective virus fusion to plasma- and intracellular- membranes, as well as to protein-free liposomes, requires the target membrane to contain anionic lipids such as bis(monoacylglycero)phosphate and phosphatidylserine. Anionic lipids act downstream of low-pH-dependent fusion stages and promote the advance from the earliest hemifusion intermediates to the fusion pore opening. To reach anionic lipid-enriched late endosomes, DEN travels through acidified early endosomes, but we found that low pH-dependent loss of fusogenic properties of DEN is relatively slow in the presence of anionic lipid-free target membranes. We propose that anionic lipid-dependence of DEN fusion machinery protects it against premature irreversible restructuring and inactivation and ensures viral fusion in late endosomes, where the virus encounters anionic lipids for the first time during entry. Currently there are neither vaccines nor effective therapies for DEN, and the essential role of the newly identified DEN-bis(monoacylglycero)phosphate interactions in viral genome escape from the endosome suggests a novel target for drug design.

## Author Summary

Dengue virus infection is a growing public health problem with up to 100 million cases annually, and neither vaccines nor effective therapies are available. To search for the ways of preventing and treating dengue infections we need to better understand their molecular mechanisms. As with many other viruses, dengue virus enters cells by fusion between the viral membrane and the membrane of intracellular vesicles (endosomes). In this work we explored the fusion stage of dengue virus entry in different experimental systems ranging from virus fusion to artificial lipid membranes to fusion inside the cells. While earlier work on dengue virus entry has focused on viral protein that mediates fusion, we found that effective action of this protein requires specific lipid composition of the membrane the virus fuses to. In effect, this lipid dependence allows virus to control intracellular location of the fusion event and, thus, the place of its RNA release by exploiting cell-controlled differences between lipid compositions of different organelles the virus travels through. The essential role of the interactions between dengue virus and its lipid cofactors during viral entry suggests that these interactions may be targeted in drug design.

## Introduction

With almost half of the world's population at risk for dengue infections, including life-threatening dengue hemorrhagic fever and dengue shock syndrome [1], the lack of vaccines and effective therapies lends urgency to the search for new targets for antiviral drugs. In this work we have focused on the membrane fusion stage of DEN infection. As many flaviviruses and alphaviruses, DEN enters mosquito and human cells by receptor-mediated endocytosis [2]–[5]. Fusion between the DEN envelope and the endosomal membrane is mediated by an envelope glycoprotein E [6]–[9] structurally similar to E protein of other flaviviruses and to E1 protein of alphaviruses such as Sindbis virus (SIN) [8], [10]. Acidification of endosomal content triggers a fusogenic restructuring of protein fusogens and fusion in flaviviruses and alvaviruses. DEN E undergoes a major conformational change that starts with the dissociation of the homodimeric form of E. Separated monomers rise from their initial positions parallel to the viral envelope to positions perpendicular to the envelope [6]–[9]. E monomers interact with the target membrane via their hydrophobic fusion loops and assemble into homotrimers that bridge the viral and target membranes. Subsequent refolding of the E trimer into its final hairpin structure, with the transmembrane domains and fusion loops at the same end of the rodlike molecules, bends the target and viral membranes towards each other and primes them for fusion [6], [8], [11]–[12].

Several observations have suggested that DEN fusion, in addition to low pH dependence, may involve as-yet-unidentified cofactors. Early research, confirmed in our preliminary experiments, found that low pH application to adjacent cells with DEN virions bound to the cell surface resulted in cell fusion for mosquito cells [13]–[15] but not for mammalian cells [13]. Furthermore, during viral entry into mammalian cells most DEN particles fuse only when they get to late endosomes/lysosomes [3]–[5], and, thus, they neither fuse nor become inactivated in early endosomes, which are expected to have pH low enough to trigger conformational changes in DEN E and induce DEN-mediated fusion between mosquito cells [15]. We suggested that in analogy to alphavirus fusion dependence on lipid cofactors (namely, cholesterol and sphyngomyelin) [16]–[19], the apparent differences in fusogenic properties of DEN towards different target membranes may reflect differences in their lipid composition. In contrast to the outer leaflets of plasma membranes of mammalian cells and to the inner leaflets of membranes of early endosomes, the outer leaflets of the plasma membranes of mosquito cells and the membranes of late endosomes of mammalian cells have high concentrations of the anionic lipids (AL) phosphatidylserine (PS) [20] and bis(monoacylglycero)phosphate (BMP) [21], respectively.

In this work, we explored the dependence of fusion of DEN, serotype 2, strain TH-36 (below referred to as “DEN”) on the lipid composition of the target membranes and found this fusion reaction to require the presence of AL. Liposomal, plasma, and intracellular membranes that did not support DEN fusion became fusion-competent upon addition of AL such as BMP and PS. The discovered dependence on AL was also observed for dengue virus serotype 2, strain New Guinea C and for dengue virus, serotype 4, strain H241. AL acted downstream of low pH-dependent stages of DEN fusion and promoted an advance of the fusion process beyond the earliest hemifusion intermediates. Endocytosed viral particles are acidified, and thus the restructuring of DEN E towards its fusogenic conformations is expected to be triggered before DEN comes into contact with the AL-containing membranes of late endosomes. For many viruses, including influenza virus [22] and SIN [17], premature activation of the viral fusion machinery by an acidic medium in the absence of a target membrane results in a quick inactivation of the machinery. In contrast, we found that DEN particles treated with a low-pH medium in the presence of AL-free target membranes retain their fusogenic properties for a relatively long time (>30 min). This unusually slow inactivation may explain the preservation of fusogenic properties of DEN for the duration of viral trafficking from early to late endosomes. We propose that the AL dependence and the delayed inactivation of the fusion machinery of DEN play an important role in defining the timing and location of the fusion stage of viral entry.

## Results

### DEN does not fuse plasma membranes of mammalian cells

**Figure 1:**
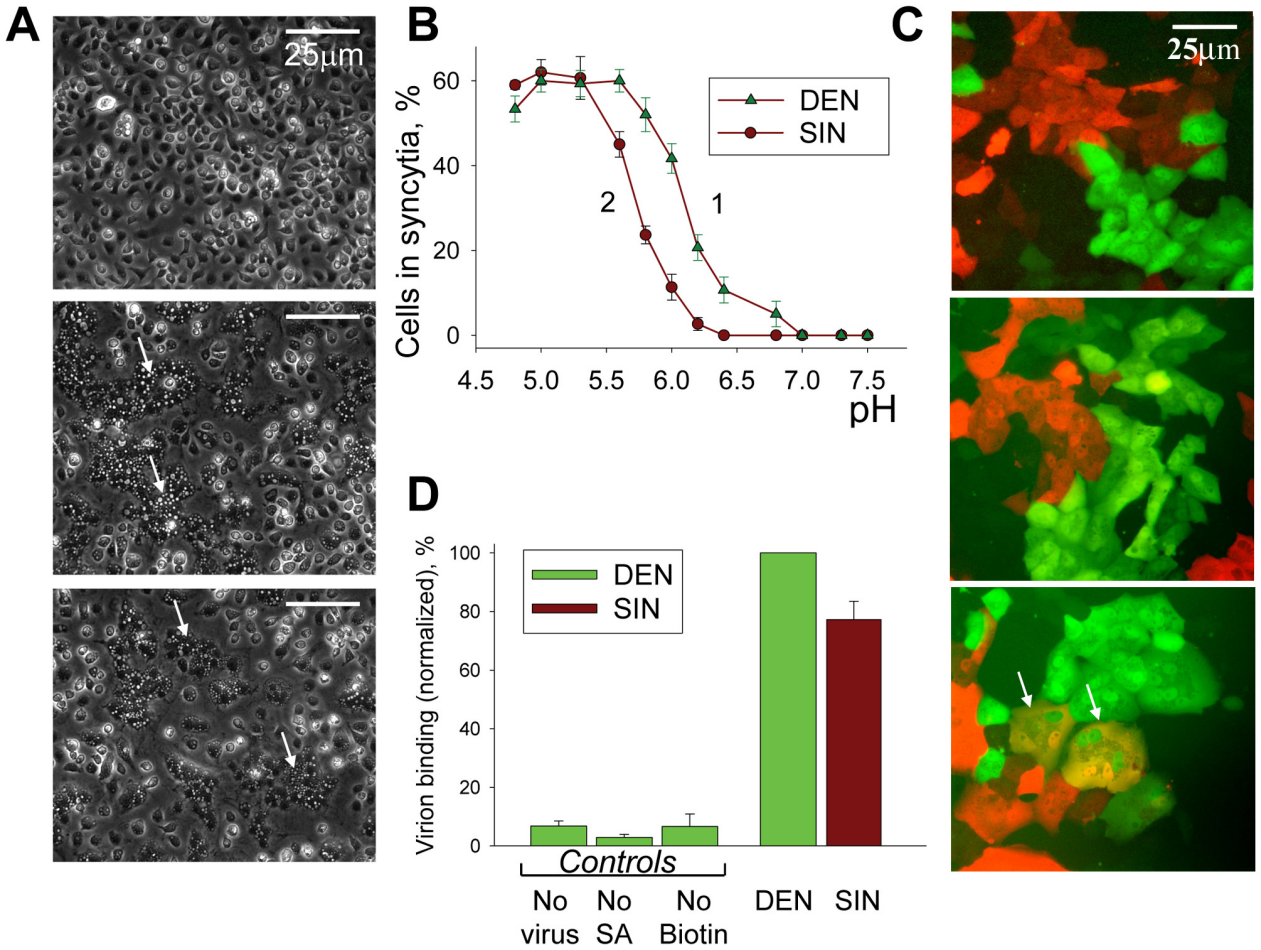
In contrast to SIN, DEN fuses mosquito cells but does not fuse mammalian cells. **A.** Fusion of mosquito cells C6/36 mediated by DEN (middle image) and SIN (image at the bottom) was triggered by a 15-min application of a pH 5.3 medium, 37°C. Images were taken after 2-h incubation in complete medium at 37°C. Arrows mark characteristic syncytia. Image at the top presents the control experiment in which pre-incubation with virions was omitted. **B.** pH dependencies of fusion between C6/36 cells mediated by DEN and SIN (triangles and circles, respectively). Fusion between cells with bound DEN- or SIN- virions was triggered at 37°C by a 15-min application of medium of a given pH. Efficiency of syncytium formation was determined by measuring a decrease in the number of mononucleated cells 2 hours later. **C.** Co-plated EGFP-expressing (green) and mRedFP-expressing (red) Vero cells were pre-incubated with DEN (middle image) or SIN (image at the bottom) and fusion was triggered by a 5-min application of a pH 5.3 medium. Double-labeled multinucleated cells (marked by arrows) were observed in the experiments with SIN but not in the experiments with DEN (image at the bottom). Image at the top presents the control experiment in which pre-incubation with virions was omitted. Images were taken 30 min after the end of the low pH application. **D.** The difference in the ability of DEN and SIN to fuse mammalian cells cannot be explained by the difference in virus-cell binding. Binding of DEN and SIN to Vero cells was evaluated using surface-biotinylated virions. Cells with associated virions were treated with streptavidin Alexa Fluor 488 conjugate and cell-associated fluorescence was analyzed by flow cytometry analysis. The bars present mean fluorescence values measured for cells incubated with biotinylated DEN (taken as 100%) or SIN and then streptavidin. “No virus” - cells were not incubated with virions but were incubated with streptavidin. “No SA” - cells were incubated with biotinylated DEN but streptavidin application was omitted. “No Biotin” - cells incubated with DEN virions, which were not biotinylated, were then incubated with streptavidin. **B & D.** The data are presented as means +/− s.d. (n≥3).

The fusogenic activity of diverse enveloped viruses is often characterized by measuring fusion between adjacent cells that is mediated by viral particles associated with the cell surface either through non-specific binding or through virus–receptor docking. For viruses that fuse in acidified endosomes, activation of viral fusogens and cell-to-cell fusion yielding syncytia is triggered by a short-term application of acidic pH. In agreement with earlier reports [13], DEN effectively fused mosquito cells C6/36 (Fig. 1A,B). Fig. 1B, curve 1 represents the pH dependence of DEN-mediated fusion of these cells and, as expected [15], already shows significant fusion at moderately acidic pH.

In striking distinction from insect cells, DEN produced in either insect or mammalian cells did not induce syncytium formation for any mammalian cells we tested (Vero, BHK21, CHO-K1, MA104, NIH 3T3, HAb2, BS-C-1, U967 (monocytes), and Raw (macrophages)) in the experiments in which cells pre-incubated with DEN were treated for 1 to 15 min with an acidic medium of pH values ranging from 4.9 to 6.5. Syncytium formation involves both the fusogen-dependent opening of fusion pores and their cell-machinery-dependent expansion, yielding a cell-size lumen [23]. We found that the absence of DEN-induced syncytia for mammalian cells reflects the inability of DEN to form fusion pores large enough to pass GFP and mRedFP, as evidenced by the lack of double-labeled cells (shown for Vero cells in Fig. 1C).

In parallel experiments, we contrasted the fusogenic activity of DEN with the well-characterized fusogenic activity of SIN, a virus that utilizes similar fusion machinery [8]. As expected, SIN effectively fused both mammalian cells and mosquito cells C6/36 (Fig. 1A; B, curve 2; and 1C; see also [24]).

We explored whether the inability of DEN to fuse mammalian cells can be explained by inefficient virus–cell-surface binding. To evaluate the binding (including non-specific receptor-independent binding), we incubated Vero cells (Fig. 1D) or CHO-K1 cells (not shown) with mildly biotinylated DEN or SIN particles, applied fluorescence-tagged streptavidin, and then carried out flow cytometry analysis (Fig. 1D). We found virus–cell binding for DEN to be somewhat higher than for SIN, the virus that effectively fuses the cells indicating that cell fusion for DEN is blocked downstream of cell-surface binding.

This conclusion was further substantiated by comparing DEN binding to C6/36 cells that were readily fusable by the virus with DEN binding to mammalian cells (MA104 cells) that did not fuse in the presence of DEN. In these experiments, we blocked internalization of DEN virions labeled with a fluorescent lipid DiD by low temperature (10°C) and quantified viral binding by measuring DiD fluorescence associated with the cells (Fig. S1). To take into account the very different sizes of C6/36 and MA104 cells, we normalized the amount of DiD fluorescence associated with the plasma membrane of the cells to the fluorescence of membrane probe NBD-tagged phosphatidylcholine (NBD-PC). This normalization is based on the assumption that NBD-PC similarly partitions into the outer leaflets of plasma membrane bilayers of C6/36 and MA104 cells and, thus, the cell-associated NBD fluorescence is proportional to the total area of plasma membranes. As shown in Fig. S1, under the conditions of our cell-to-cell fusion experiments on C6/36 cells and on MA104 cells, the ratios of DiD- and NBD- fluorescences, and thus the surface densities of DEN virions at plasma membrane, were very close. This finding argues against the hypothesis that DEN fuses C6/36 cells but does not fuse mammalian cells because of a large difference in the amounts of cell-bound virions.

To summarize, while DEN binds to and effectively infects both mosquito and mammalian cells, in our experiments it fused mosquito but not mammalian cells. The inability of DEN to fuse the plasma membranes of the virus-permissive mammalian cells indicated that the fusogenic activity of DEN depends on the target membrane. To identify the cofactors that have to be present to support DEN fusion, we concentrated on the unusual lipid composition of the late endosomal membranes that DEN fuses with to inject its RNA into cells. Using liposomes, we explored the dependence of DEN fusion on the composition of the target membrane.

### Virus-liposome fusion

**Figure 2:**
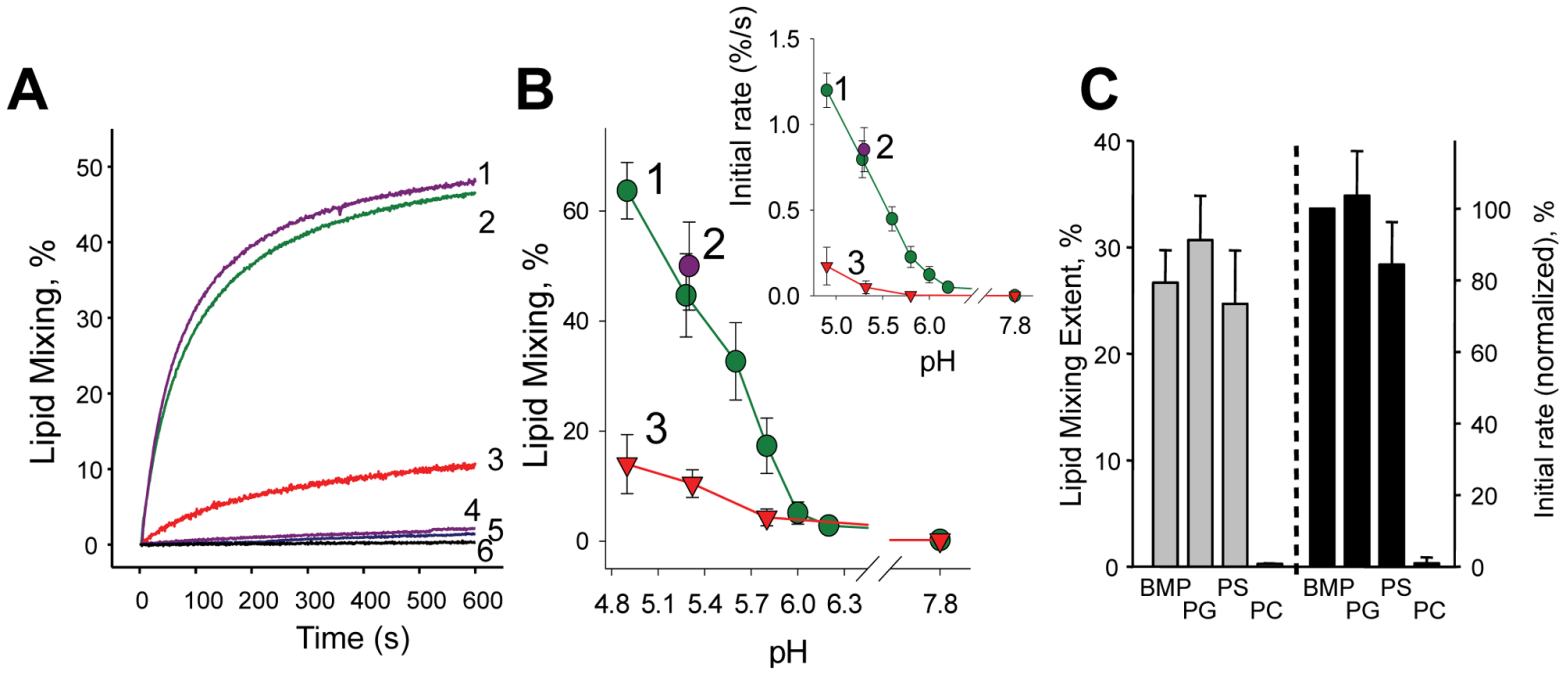
The dependence of DEN fusion to liposomes on anionic lipids. **A.** Lipid mixing between DiD-labeled DEN virions and liposomes of LEM-Chol (1); LEM (2); and PM (3) compositions (pH 5.3, 37°) assayed as DiD dequenching. 4 & 5 –LEM liposomes with DEN virions inactivated by pre-incubation at pH 4.5 or by DEPC. 6 –DEN and LEM liposomes at pH 7.8. **B.** The extent of the lipid mixing between DEN and LEM- (1); LEM-Chol- (2) and PM- (3) liposomes at different pH observed 10 min after acidification. Insert –initial rates of lipid mixing between DEN and LEM- (1); LEM-Chol- (2) and PM - (3) liposomes at different pH. **C.** Fusion between DEN virions and liposomes formed of mixtures of 70 mol % PC and 30 mol % of either BMP or PG or PS or liposomes formed of PC was measured at pH 5.3 and quantified as initial rates of lipid mixing (black bars, BMP-containing liposomes (“BMP”) taken as 100%) and the extents of the lipid mixing at t = 10 min (grey bars). The data in **B** and **C** are presented as means +/− s.d, n≥3.

To study the role of membrane lipid composition in DEN fusion, we measured low pH-induced lipid mixing between DEN particles labeled with a self-quenching concentration of a fluorescent lipid, DiD, and unlabeled liposomes of different composition. Only slow and inefficient lipid mixing between DEN and liposomes was observed for liposomes formed from lipids characteristic of the outer leaflet of the plasma membranes of mammalian cells (PM composition) (Fig. 2A,B). In contrast, we observed robust lipid mixing between DEN and liposomes containing phospholipids characteristic of late endosomal membranes (LEM composition). A much higher efficiency of DEN fusion to LEM liposomes than to PM liposomes was also observed for dengue virus serotype 4, strain H241 (Fig. S2) and for dengue virus, serotype 2, strain New Guinea C (not shown).

As expected [19], including Chol into the LEM lipid mixture (LEM-Chol composition) did not significantly change the initial rate or the extent of lipid mixing detected at t = 10 min (Fig. 2A,B). Inactivation of DEN particles by pre-incubation at pH 4.5 for 30 min (37°C) or by a histidine-modifying reagent diethylpyrocarbonate (DEPC) [25], resulted in a loss of DEN fusogenic properties (Fig. 2A). We also found that lipid mixing between DEN and LEM liposomes was inhibited by DEN monoclonal antibody 4G2 [26] (data not shown).

The inefficient fusion between DEN and liposomes containing lipids characteristic of plasma membranes contrasted with the robust fusogenic activity of SIN (Fig. S3). At pH 5.3, the initial rate and extent of lipid mixing with PM liposomes at t = 10 min for SIN were 10 fold and 3 fold higher than those for DEN. Strong lipid mixing between SIN and these liposomes is in agreement with earlier reports [17] and is also consistent with efficient fusion between alphaviruses and plasma membranes of mammalian cells (see above and [27]–[29]). Lipid mixing between SIN virions and PM liposomes developed slower than that between SIN and LEM-cholesterol liposomes but reached similar extents. Note that the rate of SIN-liposome lipid mixing detected in our experiments using the DiD dequenching assay is notably lower than that reported in [17], [30] and closer to the rates reported in [31]–[32]. This divergence most likely reflects the differences in assays used by different groups.

We hypothesized that LEM liposomes support DEN fusion because these liposomes contain an AL BMP, a specific lipid marker of multivesicular late endosomes that is present in different membrane domains of these organelles in concentrations between 20 and 70 mol% [21]. To test this hypothesis, we used liposomes of simpler compositions. While DEN did not fuse with liposomes that were formed from phosphatidylcholine (PC), liposomes that in addition to PC contained 30 mol % of either BMP or phosphatidylglycerol (PG) or PS supported lipid mixing (Fig. 2C), indicating that DEN fusion to liposomes depends on the negative charge of the target membrane rather than on a specific polar group of AL.

### Anionic lipids support DEN fusion to cell membranes

**Figure 3:**
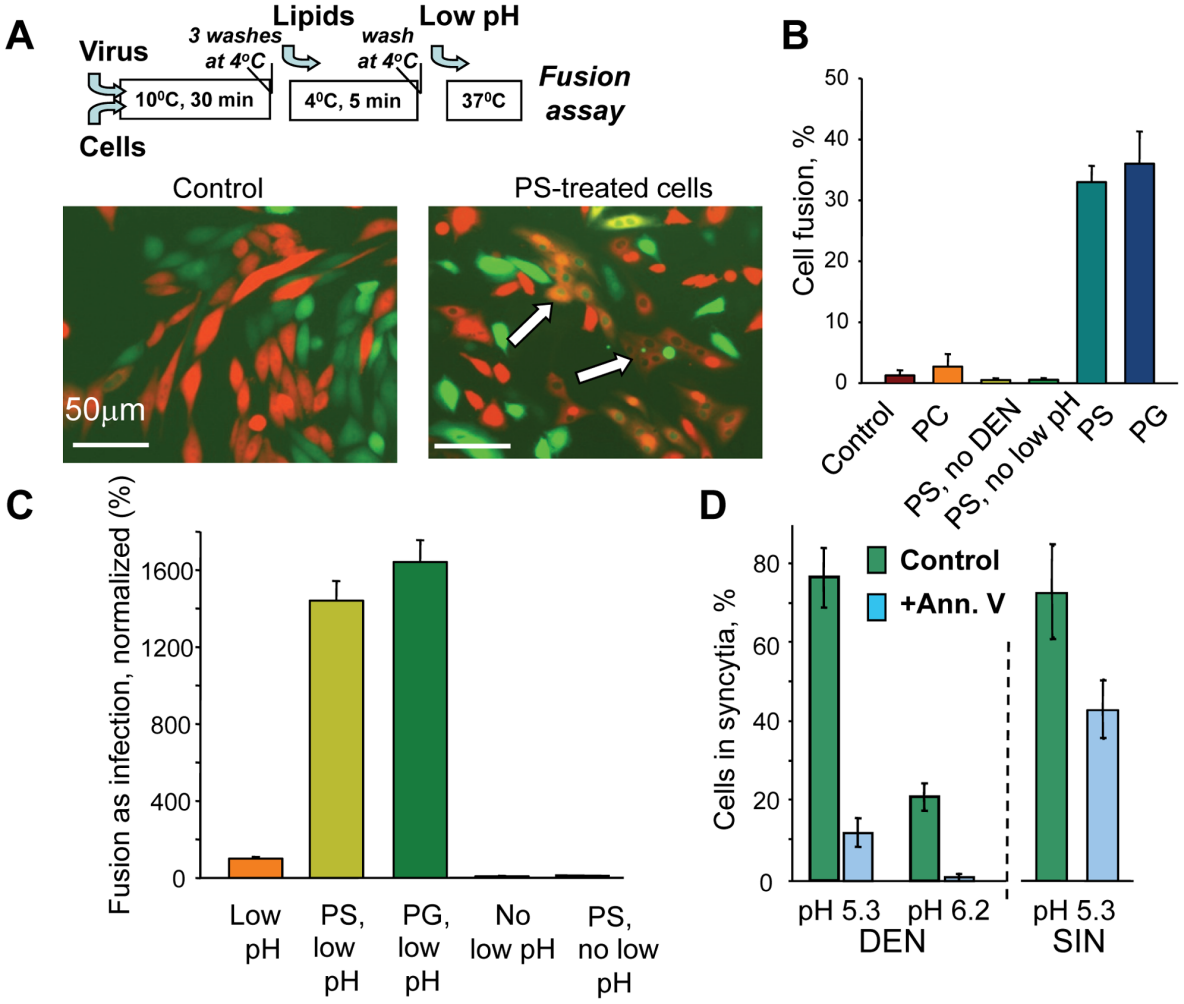
Anionic lipids promote DEN-mediated cell-to-cell fusion. **A.** Fluorescence microscopy images of co-plated EGFP-expressing (green) and mRedFP-expressing (red) CHO-K1 cells with pre-bound DEN treated with a pH 5.3 medium for 5 min (image on the left). Image on the right – Fusion of the cells treated with PS prior to a low pH application results in the appearance of fused co-labeled cells (marked by arrows). Cartoon at the top of **A** summarizes the experimental protocol used to supplement plasma membranes of CHO-K1 and other cells with anionic lipids. **B.** DEN-mediated fusion between CHO-K1 cells treated with PC, PS, or PG or with no exogenous lipids (control) immediately prior to a 5-min pH 5.3 application. (PS, no DEN) – the cells treated with PS and low pH in the absence of DEN. (PS, no low pH) – the cells with bound DEN treated with PS but not with low pH. **C.** Low pH-dependent fusion between DEN and plasma membrane of MA104 cells supplemented or not supplemented with PS or PG was evaluated as infection under conditions when endocytic entry of the virus was blocked. In the negative controls, the cells not treated (No low pH) or treated with PS (PS, no low pH) were not incubated at acidic pH. (Low pH), (PS, low pH) and (PG, low pH), – the cells not treated with exogenous lipids (taken as 100%), or treated with either PS or PG, respectively, were incubated at pH 5.3 for 5 min at room temperature. **D.** Fusion between mosquito cells C6/36 mediated by either DEN or SIN at pH 5.3 and by DEN at pH 6.2 is inhibited by blocking AL at the cell surface with annexin V (blue bars). (green bars) – no annexin. Note that annexin V inhibition of DEN-mediated fusion of C6/36 cells at pH 5.3 is much stronger than that for SIN-mediated fusion. **B**, **C**, **D**. The data are presented as mean +/− s.d, n≥3.

We then tested whether the inability of DEN to fuse mammalian cells can be explained by the lack of AL in the outer leaflet of the plasma membrane. We incubated CHO-K1 cells with the virus; then, after a wash, we placed the cells at 4°C and treated them with exogenous AL, PS. After a wash, we applied a low-pH medium at room temperature to trigger fusion. DEN effectively fused PS-treated cells but did not fuse untreated cells (Fig. 3A,B). While PS added to the outer leaflet of plasma membranes of mammalian cells undergoes inward transmembrane redistribution mediated by ATP-dependent aminophospholipid translocases [33], at the time of low pH application significant percentage of exogenous PS remained in the outer leaflet, as evidenced by its accessibility for extraction with bovine serum albumin, BSA (data not shown). Furthermore, DEN fusion of mammalian cells was similarly supported by addition of PG, an AL that is not an aminophospholipid (Fig. 3B). In control experiments, DEN did not mediate fusion between CHO-K1 cells if cell membranes were not supplemented with exogenous lipid or were supplemented with PC (Fig. 3B). In similar experiments carried out with SIN, we found cell fusion mediated by this virus to be almost unaffected by addition of either PS or PG (Fig. S4).

Since AL were applied only after removal of unbound DEN, the dramatic increase in fusion after PS treatment cannot be explained by a better DEN binding. We directly verified that cells treated and untreated with PS carried similar amounts of DEN particles in experiments with fluorescence-labeled viral particles. CHO-K1 cells were incubated with DiD-labeled virus at 10°C and washed. The temperature was lowered to 4°C and cells with associated virus were either treated or not treated with PS and then solubilized with 1% Triton X-100 at room temperature to dequench the DiD. The levels of fluorescence measured for samples from PS-treated and untreated cells were statistically indistinguishable.

As for many other enveloped viruses, fusion of DEN to the plasma membrane can be indirectly evaluated by measuring cell infection caused by low pH-induced fusion at the cell surface under conditions where acidification of the endosomes, essential for the biologically relevant entry pathway, is blocked [34]. We found the application of this fusion-infection assay (FIA) to DEN and mammalian cells (MA104, Vero and BHK21) to require a very high concentration of virions: 300 infectious units/cell (at least 10 times higher than the number of infectious viral particles per cell we had to use in FIA for mosquito C6/36 cells, data not shown). In this setting, FIA, while indirect, may be a very sensitive assay for fusion since fusion of a single virion out of hundreds may result in infection. As for DEN-mediated cell-to-cell fusion, the anionic lipids (PS or PG)-supplemented MA104 cells demonstrated much higher (15-fold) levels of fusion in FIA (Fig. 3C). Note that DEN-dependent fusion of PS-treated cells was still dependent on low pH. We observed a similar promotion of fusion-infection for Vero and BHK21 cells (data not shown).

The importance of AL for DEN-plasma membrane fusion was further confirmed in the experiments on mosquito cells. As mentioned above, C6/36 cells were reported to expose unusually high concentrations of AL at their surface [20]. In agreement with this study, we found a much higher cell-surface labeling with R-phycoerythrin -tagged annexin V for C6/36 than for MA104 cells (Fig. S5). Blocking PS and perhaps other AL [35] exposed at the surface of C6/36 cells with annexin V inhibited DEN-mediated fusion between these cells (Fig. 3D). While SIN-mediated fusion between C6/36 cells was also inhibited by annexin V perhaps because of a steric hindrance, inhibition of the DEN-fusion was much stronger. These findings support the hypothesis that the known ability of DEN to fuse C6/36 cells reflects the elevated concentrations of externalized AL in their membranes.

In brief, as with DEN-liposome fusion, the efficiency of DEN fusion to plasma membranes correlates with the accessibility of AL.

### Both DEN fusion within the endocytic pathway and viral infection depend on anionic lipids

**Figure 4:**
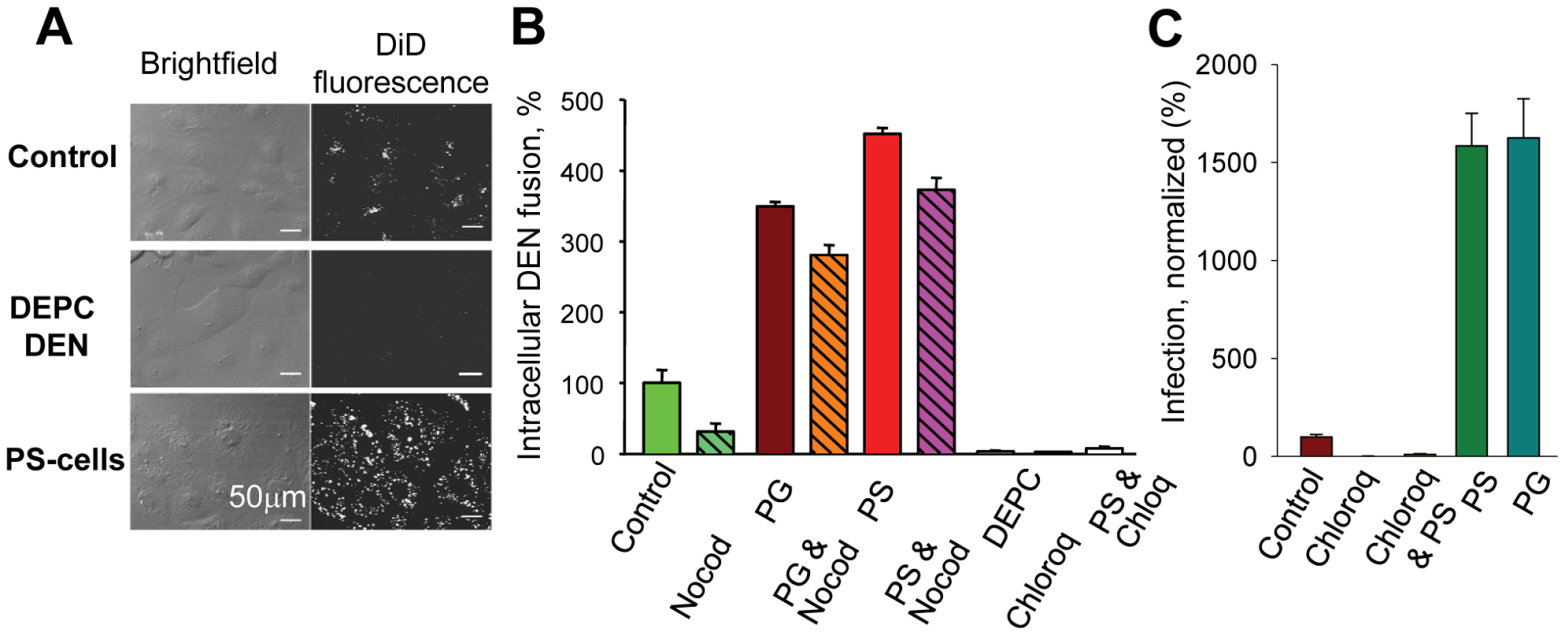
Anionic lipids promote DEN-mediated intracellular fusion and viral infection. **A.** An increase in the cell fluorescence upon fusion of DiD-labeled DEN within the endocytic pathway of MA104 cells. Images were taken 40 min after raising the temperature to allow internalization of the virus for PS-treated cells (PS-cells), untreated cells (Control) and untreated cells with DEPC-inactivated DEN (DEPC DEN). **B.** Intracellular DEN fusion was quantified as the mean total fluorescence per imaging field for: untreated MA104 cells (control, taken as 100%); cells pre-treated with either nocodazole (Nocod) or chloroquine (Chloroq); cells with DEPC-inactivated virus (DEPC); cells treated with PG (PG), cells pretreated with nocodazole and then treated with PG (PG&Nocod); cells treated with PS (PS), cells pretreated with chloroquine or nocodazole and then PS (PS & Chloroq and PS&Nocod). **C.** Promotion of DEN infection for MA104 cells treated with PS (PS) or PG (PG). Control – no exogenous lipids, taken as 100%. DEN infection of the cells treated or not treated with PS is inhibited by chloroquine (Chloroq & PS and Chloroq, respectively). **B**, **C.** The data are presented as mean +/− s.d, n≥3.

The effects of AL on fusion events during viral infection were studied in MA104 cells and BS-C-1 cells with pre-bound DEN labeled with DiD in a self-quenching concentration. Viral fusion events along the endocytic pathway diluted DiD and, thus, significantly increased cell fluorescence (Fig. 4A and S6). While first fusion events were observed less than 5 min after the rise in temperature to 37°C, most viral particles fused only at later times, with a median waiting time of ∼15 min (Fig. S6), consistent with an earlier study [3]. As expected, no intracellular fusion was observed when endosomal acidification was blocked by chloroquine or when DEN was DEPC-inactivated (Fig. 4A,B). In agreement with reports suggesting that DEN fusion reactions take place in late endosomes [3]–[5], we found that microtubule-depolymerizing nocodazole known to disrupt endosomal trafficking from early to late endosomes/lysosomes [36] inhibits intracellular fusion of DEN. When cells with pre-bound DEN were treated with AL (5-min, 4°C) before warming up to 37°C, we observed a dramatic increase in the average cell fluorescence (Fig. 4A,B). As for untreated cells, the increase in fluorescence of PS-treated cells was inhibited by chloroquine. The increase in DiD fluorescence reflects a higher efficiency of DEN–endosome fusion and suggests that in the AL-treated cells DEN fuses in early endosomes that normally would not have AL. This conclusion was further substantiated by the finding that intracellular fusion of DEN in AL-treated cells was much less sensitive to inhibition of endosomal trafficking by nocodazole than in untreated cells (Fig. 4B). Promotion of intracellular fusion for PS-supplemented cells was also observed for dengue virus of serotype 2, strain New Guinea C (not shown).

One may expect the delivery of DEN virions to late endosomal compartments that support viral fusion to be blocked by a dominant negative Rab7 (DN Rab7) that disrupts late endosomal*/*lysosomal biogenesis. Indeed, at least for some strains of DEN, DN Rab7 inhibits the infection of mammalian cells and DEN fusion [3]. As expected, we found that EGFP-tagged DN Rab7a S22N expression inhibits intracellular fusion of DiD-labeled DEN in MA104 cells, as evidenced by a much lower DiD fluorescence associated with DN Rab7-expressing cells detected by the EGFP fluorescence (Fig. S7A). Treating the cells with PS alleviated the DN Rab7 inhibition, as evidenced by the appearance of cells displaying both DiD- and EGFP- fluorescence (Fig. S7B). These findings further substantiate our hypothesis that DEN delays its fusion until entry into late endosomes because of the AL-enriched lipid composition of their membranes and does not fuse in early endosomes because these organelles normally do not have anionic lipids.

Since fusion is an early stage of DEN infection, promotion of DEN fusion in endosomal compartments may promote DEN infection. We extended our work from fusion assays to infection analysis and explored the effects of AL on the physiologically-relevant pathway of infection via endocytosis. A strong (∼16-fold) promotion of infection for cells supplemented with PS or PG (Fig. 4C) indicates that the AL dependence of DEN fusion results in a corresponding dependence of viral infection.

### Inactivation of DEN fusion machinery

**Figure 5:**
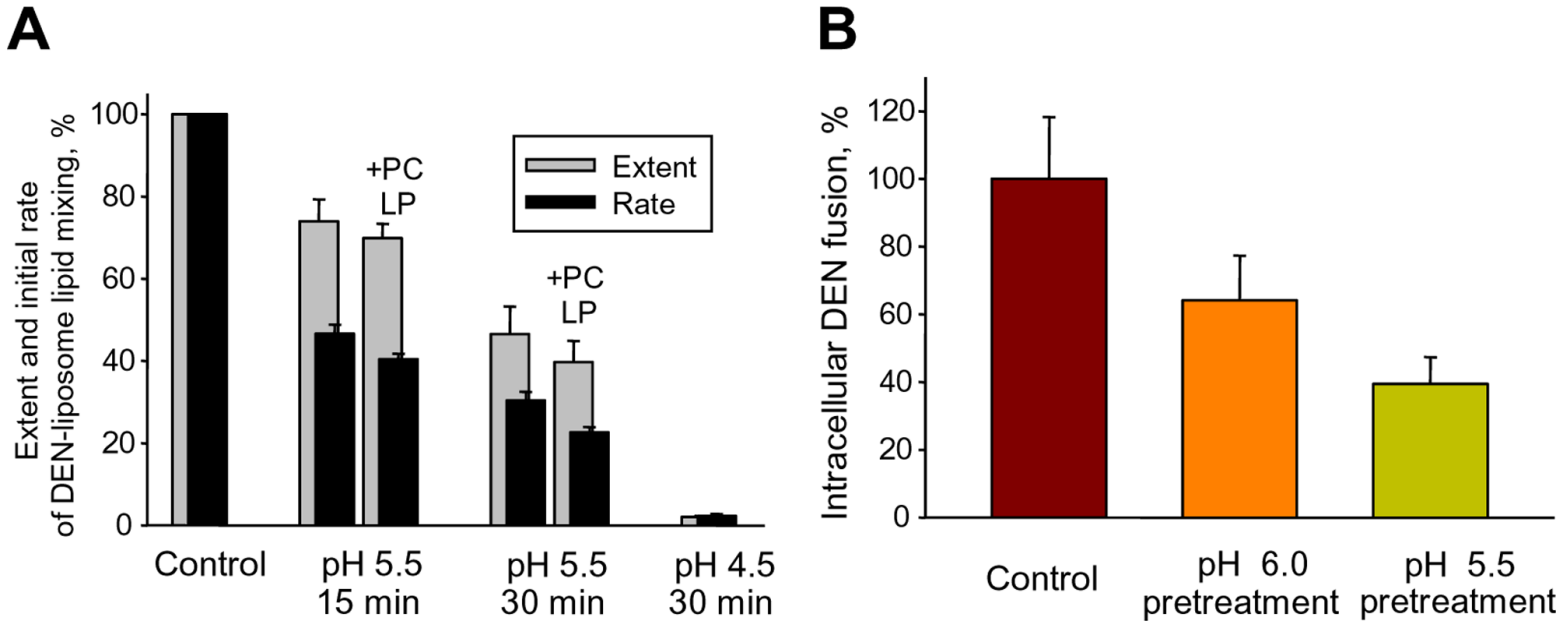
Slow inactivation of fusion machinery of DEN virions acidified in the absence of AL-containing membranes. **A.** DiD-labeled DEN virions were pre-incubated at pH 5.5 for 15 or 30 min in the absence of liposomes or in the presence of PC liposomes (+PC LP); or at pH 4.5 for 30 min in the absence of liposomes. After low pH pre-treatment, virions without re-neutralization were mixed with LEM liposomes at pH 5.5. The initial rates of lipid mixing (black bars) and the extents of lipid mixing at t = 10 min (gray bars) were normalized to those between DEN and LEM liposomes observed without low pH-preincubation (Control). **B.** MA104 cells with pre-bound DiD-labeled DEN were pre-treated or not pre-treated at 37°C with pH 6.0 or 5.5 medium (15 min, 37°C). Low pH application was followed by a 40-min incubation in the complete medium at 37°C. Viral fusion events along the endocytic pathway were detected as an increase in the mean total fluorescence per imaging field. Control – no low pH pre-treatment, taken as 100%. **A**, **B**. The data are presented as mean +/- s.d. (n = 3).

Conformational changes of DEN E and DEN fusion with insect cells are already triggered at the moderately acidic pH (∼pH 6.0) (Fig. 1B) that the endocytosed virus is expected to encounter in early endosomes. While premature activation of most viral fusogens inactivates them [37], inactivation of DEN has to be relatively slow to keep viral fusion machinery functional until the virus reaches late endosomes. Indeed, we found that more than half of DEN particles remained fusogenic towards LEM-liposomes after a 15-min pre-incubation at pH 5.5 either in the absence of the target membranes or in the presence of fusion-incompetent PC liposomes (Fig. 5A). Low pH exposure of DEN at the surface of MA104 cells resulted in similarly slow inhibition of subsequent intracellular fusion (Fig. 5B). Since most of the endocytotic cargo reaches late endosomes within 15 min [38], we concluded that the inactivation of DEN at acidic pH is slow enough to preserve the virus's fusogenic properties during its trafficking from early to late endosomes.

### The lack of anionic lipids blocks DEN fusion downstream of low-pH-dependent stages and restricted hemifusion intermediates

**Figure 6:**
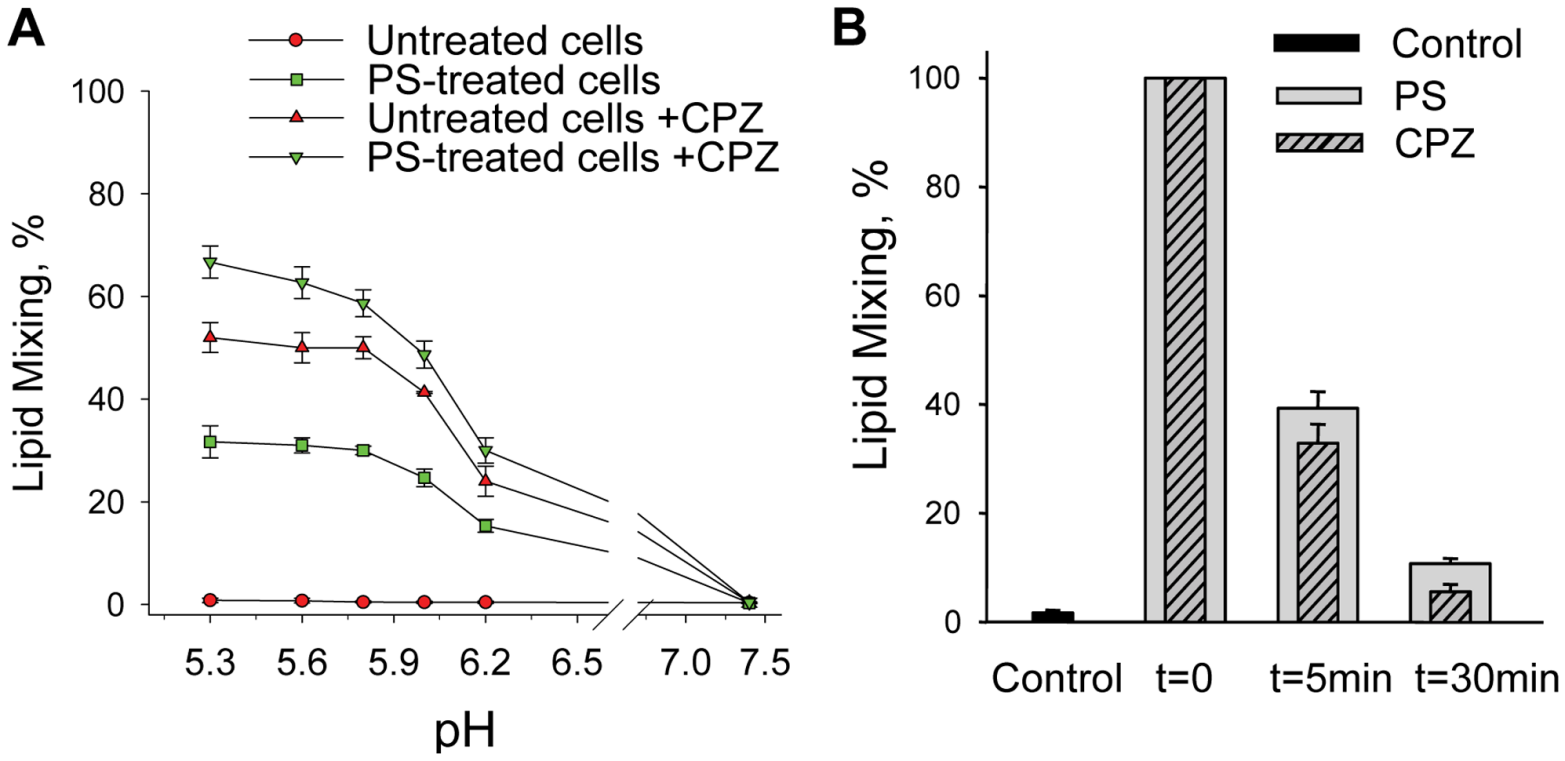
The lack of anionic lipids blocks fusion advance beyond restricted hemifusion intermediates. **A.** pH dependence of the DEN-mediated fusion between HAb2 cells and PKH26-labeled RBC for the cells treated or not treated with PS and/or with CPZ. Fusion was triggered by a 5 min application of a given pH and assayed as a percentage of HAb2-DEN-RBC complexes demonstrating lipid mixing. HAb2-DEN-RBC complexes were treated with neither PS nor CPZ (untreated cells); with PS immediately prior to low pH application (PS-treated cells); with CPZ pulse immediately after the end of low pH application (untreated cells + CPZ); with PS immediately prior to low pH application and with CPZ pulse immediately after the end of low pH application (PS-treated cells + CPZ). **B.** The loss of ability of AL added after low pH pulse to support DEN-mediated fusion correlates with dissociation of DEN E-formed restricted hemifusion intermediates. CPZ pulse (striped bars) or PS (gray bars) were applied immediately after the end of a 5-min incubation of HAb2-DEN-RBC complexes at pH 5.3 medium (t = 0, taken as 100%); 5-min or 30- min later. Control –HAb2-DEN-RBC complexes treated with neither CPZ nor PS. **A**, **B** – the data presented as mean +/- s.d., n≥3.

Which of the stages of the fusion pathway mediated by DEN E protein depends on the AL presence in the target membranes? Diverse fusion processes start with a local merger of contacting leaflets of two membranes, a stage referred to as hemifusion [39]. However, lipid flow through the earliest hemifusion intermediates may be restricted by the proteins surrounding the fusion site [39]. These ‘restricted hemifusion’ intermediates (RH) can be transformed into complete fusion with chlorpromazine (CPZ). Inverted-cone shaped CPZ preferentially partitions into the inner leaflets of cell membranes and breaks hemifusion structures composed solely of these leaflets [40]. To test whether DEN E forms RH intermediates, we used the experimental system of HAb2 cells with pre-bound human red blood cells (RBC) [41]–[42] utilized in earlier studies on RH [43]. HAb2 cells express HA0, an uncleaved form of influenza hemagglutinin that mediates very tight binding between HAb2 and RBC but is fusion-incompetent [43]. We allowed DEN to bind to HAb2 cells for 30 min at 10°C, then added RBCs labeled with a fluorescent lipid PKH26 and 15 min later washed out unbound virus and RBCs. Low pH application to HAb2–DEN–RBC complexes yielded no lipid mixing, unless the cells were pre-treated with PS (Fig. 6A). However, robust fusion was observed for cells not treated with PS when low pH pulse was followed by an immediate application of CPZ indicating that DEN-mediated HAb2-RBC fusion was blocked at the stage of RH intermediates.

The extents of lipid mixing gradually decreased when we extended the time interval between the end of low pH application and the CPZ pulse, indicating that RH formed by DEN E dissociated with time (Fig. 6B). This is similar to RH in fusion mediated by influenza and SIN viruses [43]. Likewise, 40% of cell complexes developed lipid mixing when PS was applied 5 min after the end of a 1-min pulse of pH 5.3, with much lower lipid mixing observed when PS was applied 30-min after the end of the low pH pulse. These findings indicated that pre-lipid-mixing fusion intermediates formed by low-pH-conformations of DEN E advanced to yield lipid mixing in the presence of AL independently of low-pH but dissociated with time in the absence of AL.

To summarize, DEN-AL interactions facilitate the transition from RH to more advanced fusion intermediates. The AL dependence of this transition distinguishes fusion machinery of DEN from that of SIN. As expected [43], SIN effectively mediated lipid mixing between RBC and HAb2 cells not treated with PS and CPZ application strongly promoted SIN-mediated fusion only for suboptimal pH showing that for pH≤5.8 most of the RH intermediates advanced to yield lipid mixing without CPZ application (Fig. S8).

## Discussion

This study developed from our analysis of the intriguing finding that while DEN effectively infects mammalian cells, it has a very low level of fusogenic activity towards the plasma membranes of these cells. We hypothesized that the late endosomal/lysosomal membranes that DEN fuses with during infection [3]–[5] contain cofactors for DEN fusion machinery missing in the plasma membrane. In our search for these cofactors, we have focused on the unusual lipid composition of the late endosomal membrane and found that the AL highly enriched in these membranes are indeed required for low-pH-dependent DEN fusion. Since in mammalian cells DEN comes into contact with AL-enriched membrane leaflets only in late endosomes, the AL dependence of DEN fusion likely determines the timing and the place of the release of the viral RNA into the cytosol.

### The mechanism of AL dependence

All membrane fusion reactions are expected, and in many cases shown, to depend on the lipid composition of the membranes. Some dependencies are conserved among fusion processes driven by very diverse fusogens [39]. In contrast, the essential role of AL for DEN fusion is not conserved even within the same class (II) of viral protein fusogens. In spite of similarities between the structures of the fusogens utilized, another flavivirus, TBE, and alphaviruses such as SIN effectively fuse with AL-free target membranes [44]–[45]. Interestingly, although it is AL-independent, the fusion of alphaviruses including SIN does require the specific lipids cholesterol and sphingomyelin as cofactors for the protein machinery [46]–[48], and neither of these lipids is required for DEN fusion.

The specific mechanisms by which DEN E interactions with AL control E restructuring and fusion remain to be clarified. To start with, the local pH near a membrane containing AL is lower than the pH in the bulk of the solution because of the accumulation of protons near the negatively charged lipid headgroups. The changes in ion concentration at a charged surface are described by the Gouy-Chapman theory that yields the relation between the surface charge density, the electrostatic potential, the ionic strength of the solution and ion distributions near charged membranes [49]. An estimate based on the Gouy Chapman theory suggests that for a bilayer containing 30 mol% AL in 100 mM NaCl buffer, the apparent pH dependence of fusion may be shifted to a less acidic pH by up to 0.7 units. However, while DEN fusion with liposomes containing 30 mol% PS was already observed at pH 6.8, there was almost no lipid mixing for AL-free PC liposomes even at pH 4.5 (not shown). Thus, the AL-induced shift of local pH does not fully explain the role of AL as a prerequisite for DEN fusion. Note that pH dependences of fusion observed for dengue virus in different assays somewhat differ (see for instance, Fig. 1B and Fig. 2B). These differences likely reflect different numbers of activated fusion proteins required to reach detectable fusion stages for different target membranes.

**Figure 7:**
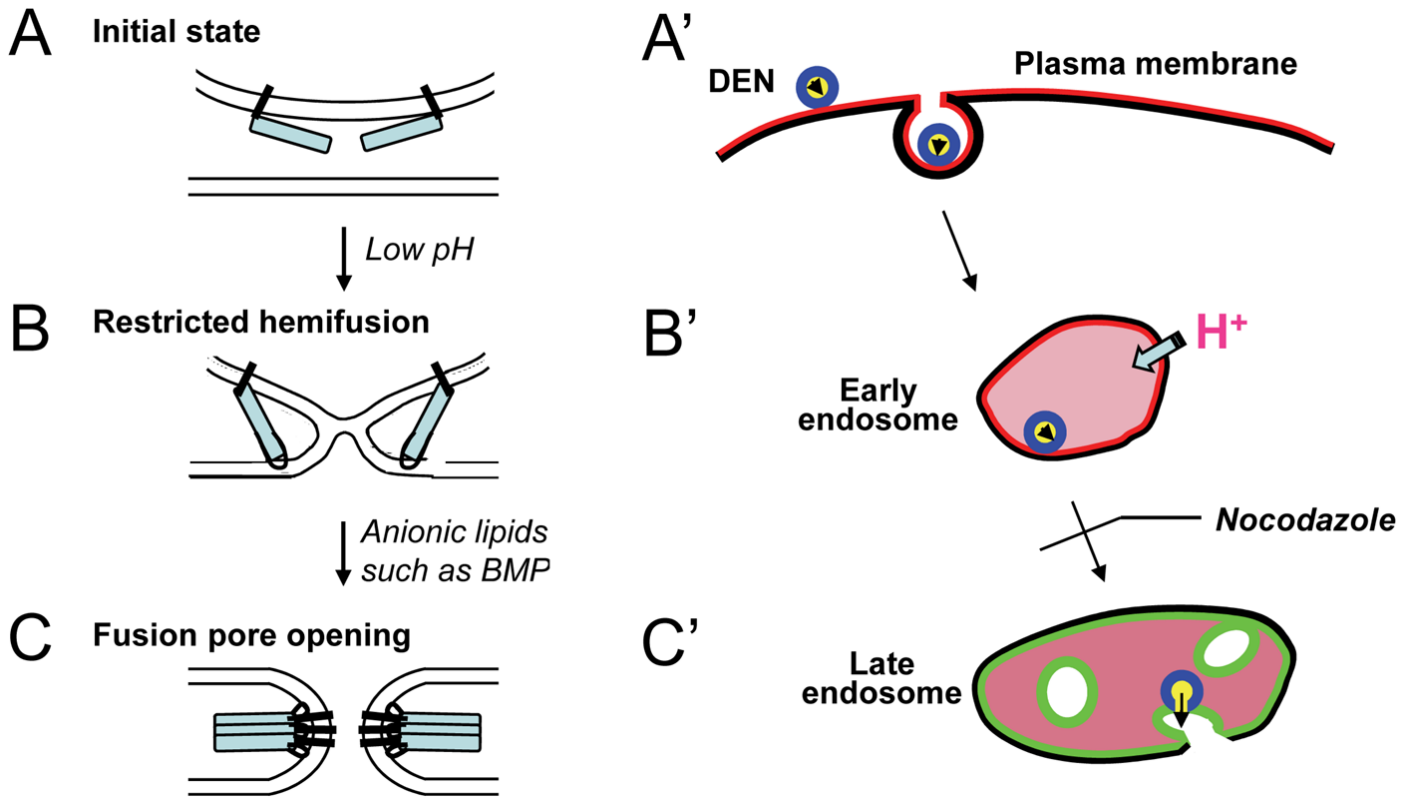
The schematic diagram that illustrates the proposed mechanism of DEN entry. Left panel (**A**, **B**, **C**) illustrates enlargement of the fusion intermediates corresponding to distinct stages of viral entry shown in the right panel (A',B', C'). **A.** Initial state of membrane contact. DEN E lays along the surface of the viral envelope. **B.** Acidification in the presence of AL-free target membrane (for instance, in the early endosome) destabilizes the E dimers and allows E monomers to interact with the target membrane. These interactions result in formation of the earliest hemifusion intermediates that do not support lipid mixing between the membranes. **C.** Protein E interaction with AL-enriched target membrane (for instance, late endosomal membrane) allows productive insertion of the fusion loop of E required for homotrimerization of E protein. Homotrimers of E drive the transition from restricted hemifusion to complete fusion. The panel on the right (**A'**, **B'**, **C'**) summarizes the proposed pathway of the DEN entry. While endocytosed viral particles in early endosomes are already exposed to environment acidic enough to trigger conformational changes in DEN E (**B'**), most of the virions neither inactivate nor advance in fusion beyond restricted hemifusion until microtubule-dependent trafficking delivers them into the late endosomal environment and, for the first time during entry, virions come into contact with an AL-enriched target membrane (**C'**). Virion fusion to the AL BMP-enriched membrane of late endosomes (shown in green) delivers viral RNA to its translation-replication sites at pre- and post-lysosomal vacuoles.

In the fusion pathway mediated by several viruses, RH is followed by more-energy intensive stages that culminate in opening of an expanding fusion pore [39], [43]. While DEN forms the RH intermediates in the absence of AL, the later stages of DEN fusion require interactions between E protein and AL. In alphavirus fusion, the lipid cofactor cholesterol promotes the insertion of E1 fusion loops into the target membrane and formation of functional E1 homotrimers [47]. In analogy to this mechanism, we propose that the completion of DEN fusion requires E assembly into stable trimers dependent on interactions between the fusion loop of DEN E and AL in the target membrane (Fig. 7). Indeed, for a synthetic peptide representing the fusion loop region of DEN peptide, insertion into the lipid bilayer and peptide–peptide interactions at the bilayer surface are promoted in the presence of AL [50].

### DEN fusion assays

To facilitate the development of antivirals targeting the fusion stage of DEN entry it is important to have a reliable quantitative approach that may be used in a high throughput *in vitro* screening. In the most recent studies [11], [51], DEN fusion to liposomes has been detected by exposure of the viral core protein to liposome-encapsulated trypsin. A successful application of this approach (first developed for another virus in [52]) to DEN is an important advance. However this approach is difficult to quantify and still needs to be validated by excluding the possible role of leakages [52] that may accompany DEN-target membrane interactions at acidic pH [51]. For many viruses, including SIN and some flaviviruses, fusogenic activity can be conveniently monitored by measuring lipid mixing between viruses and liposomes [16]–[17], [19], [30]–[32], [44], [52]–[55]. However, development of a lipid mixing assay for DEN fusion has proved to be surprisingly challenging [56]. Our work explains the very low efficiency of lipid mixing between DEN virions and AL-free liposomes [11] and describes a simple fluorescence dequenching assay of fusogenic activity of DEN towards AL-containing liposomes.

In addition to a lipid mixing assay that characterizes fusion between DiD-labeled DEN and liposomes by measuring DiD dequenching, we also used DiD-labeled virions to develop the intracellular DEN fusion assay. Our assay is based on earlier work [3], [57] that elegantly explored the DEN entry pathway in living cells by following a single DiD-labeled DEN particle using real-time fluorescence microscopy. In our approach, instead of detailed characterization of the time course and localization of the DEN fusion events for ∼50–100 virions achieved in [3], [57], we have focused on developing a much simpler approach characterizing intracellular DEN fusion as averaged over thousands of virions and cells. We expect our quantitative assays of DEN fusion within cells and with liposomes to be of help in screening for potential anti-DEN drugs.

### The biological relevance of AL dependence

Viruses have developed different strategies to prevent the premature release of the conformational energy of their protein fusogens that would result in irreversible “discharge” or in undesirable fusion. For many viruses, including DEN, which utilize low pH-dependent fusogen proteins, these proteins are synthesized in an inactive form and then are converted to a mature fusion-competent form by proteolytic cleavage of the fusogen itself or an accessory protein [6]–[7], [10]. However, viral fusogens such as DEN E that can be activated at pH values close to neutral may need additional mechanisms for avoiding premature release of the conformational energy stored in the proteins. Our finding that DEN neither fuses nor rapidly inactivates in the absence of AL-containing target membranes suggests that AL dependence of DEN prevents functional inactivation of the virus until it reaches AL-enriched late endosomes (Fig. 7). RH connections provide an additional receptor-independent docking mechanism that holds viral and endosomal membranes in tight contact. Within multivesicular endosomes, the virus can fuse either to the limiting membrane or to the internal vesicles that contain the highest concentration of AL BMP [21]. In the latter case, viral RNA release requires an additional fusion event: BMP-dependent but likely DEN E-independent back-fusion between the internal vesicle and the limiting membrane. Back-fusion has been proposed as a mechanism for endosome-to-cytosol transport of RNA of vesicular stomatatis virus ([58], but see [59]).

Our work concentrates on fusion mediated by DEN serotype 2, strain TH-36. While experiments on virus/liposome lipid mixing for dengue virus of serotype 4, strain H241 (Fig. S2) and experiments on virus/liposome lipid mixing and intracellular fusion for dengue virus of serotype 2, strain New Guinea C suggest that these viruses have a similar dependence on AL, DEN virions of different serotypes and strains may use alternative pathways for their entry into mammalian cells [60]–[61]. Future research will clarify the applicability of the AL-dependent mechanism of timing viral fusion to the entry into late endosomes for different serotypes/strains of DEN.

To summarize, the AL dependence of DEN fusion identified in our work suggests a novel mechanism allowing viruses to exploit cell-controlled changes in membrane lipid composition. In this mechanism, internalized virus uses the specific lipid composition of late endosomes highly enriched in AL as a way of timing fusion to deliver viral RNA to its translation-replication sites effectively. We hope the assays developed in this study to directly characterize DEN fusion will help in developing antivirals including those targeting DEN-AL interactions to block or prematurely activate DEN E refolding. Furthermore, interactions between DEN E and AL may have implications for the pathogenesis of dengue hemorrhagic fever, which is characterized by activation of endothelial cells and extracellular exposure of PS.

## Materials and Methods

### Cells

Vero, MA104, BHK21, BS-C-1, CHO-K1, RAW 264.7, U967, and NIH3T3 cells (American Type Culture Collection (ATCC), Manassas, VA), and HAb2 cells (a kind gift of Dr. Judith White, University of Virginia), a line of NIH 3T3 cells stably expressing A/Japan/305/57 influenza hemagglutinin (HA) in the immature fusion-incompetent HA0 form [41] were grown in Advanced DMEM medium (ADMEM) supplemented with 10% fetal bovine serum, 25 mM HEPES, 2 mM glutamine, and antibiotics (complete medium) at 37°C and 5% CO_2_. *Aedes albopictus* C6/36 (American Type Culture Collection, Rockville, MD) were cultured in the complete medium at 28°C and 5% CO_2_. To facilitate detection of cell fusion events by coplating differently labeled cells, we developed Vero, CHO-K1, and BHK21 cells stably expressing either EGFP or mRedFP according to the standard procedure using pEGFP and pmRFP plasmids (kind gifts of Dr. Eugene Zaitsev, NIH, NICHD).

Human red blood cells (RBC) were freshly isolated from whole blood obtained from the National Institutes of Health (Bethesda, MD) blood bank and labeled with a fluorescent lipid PKH26 (Sigma, St. Louis, MO), as described in [62]–[63].

### Viruses

If not stated otherwise, we used dengue virus of serotype 2, strain TH-36. In some experiments we used dengue virus of serotype 2, strain New Guinea C and dengue virus of serotype 4, strain H241. All viruses were purchased from ATCC. We propagated DEN by inoculating monolayers of C6/36 cells or Vero cells in complete medium in the presence of 0.005% Pluronic F-127 (Invitrogen, Eugene, OR) at a multiplicity of infection (MOI) of 0.1. DEN particles released from the cells were harvested 5 days postinfection and cleared from cell debris by means of low-speed centrifugation. Working virus stocks with titers between 10^7^ and 10^8^ infectious units (IU)/ml were kept at 4°C for less than two weeks. SIN strain AR-339 (ATCC) was used for infection of Vero or C6/36 cells with a low multiplicity of infection to propagate the working virus stocks with titer between 10^8^–10^9^ IU/ml. Virus was collected 24-hours post-infection. DEN and SIN were titrated for Vero cells using a fluorescent focus assay.

Viruses were concentrated by overnight centrifugation (SW28 rotor, 21 K rpm, 4°C) on a 55% cushion of Optiprep Density Gradient medium (Sigma, St. Louis, MO) buffered with 20 mM Tricine-HCl and 140 mM NaCl, pH 7.8 (buffer TrN) supplemented with 0.005% Pluronic F-127.

To label viral particles with a self-quenching concentration of a fluorescent lipid, we mixed 10 µl of a 1 mM DiD solution from a Vybrant cell-labeling kit (Molecular Probes, Eugene, OR) with 1 µl of 10% Pluronic F-127 and bath-sonicated the dispersion for 5 min. Freshly prepared DiD dispersion was injected into 1 ml of virus stock (approximately 4×10^8^ IU) under intensive vortexing. The mix was incubated for 30 min at room temperature and then for 2 hr at 4°C. Labeled virus was purified from unincorporated dye and from non-viral membranes and proteins by centrifugation (SW55 rotor, 1.5 h, 53 K rpm, 4°C) on a 40%–25%–20%–15% step gradient of Optipep density medium in TrN supplemented with 0.005% Pluronic F-127. We collected the band between 20% and 25% densities containing DiD-labeled virus. BSA (final concentration 1%) was added to stabilize the preparation. Labeled virus was used within 3 days. Before experiments, the viral suspension was passed through a PES Millipore 0.22 µm filter to remove viral aggregates. In fluorescence microscopy examination, the number of DiD-labeled spots practically coincided with the number of fluorescent spots observed when viral particles (DEN or SIN) were visualized by immunofluorescence with DEN antibody (MAB 8705) or SIN antibody (Sindbis Hyperimmune Ascetic Fluid). DiD-labeled virus retained infectivity, but with an approximately two-fold titer decrease.

### Liposomes

All lipids used: Chol, PC (1,2-dioleoyl-*sn*-glycero-3-phosphocholine); PE (1,2-dioleoyl-*sn*-glycero-3-phosphoethanolamine); SPM (N-oleoyl-D-erythro-sphingosylphosphorylcholine); PI (L-α-phosphatidylinositol, Soy); BMP (bis(monooleoylglycero)phosphate, S,R Isomer); PS (1,2-dioleoyl-*sn-*glycero-3-phospho-L-serine); and PG (1,2-dioleoyl-*sn*-glycero-3-phospho-(1′-*rac*-glycerol)) were purchased from Avanti Polar Lipids, Alabaster, AL. Liposomes were formed from the following lipid mixtures: **PM composition**: PC, phosphatidylethanolamine (PE), sphingomyelin (SPM), cholesterol (Chol) in a molar ratio of 4/1/0.5/4.5; **LEM composition**, PC/PE/phosphatidylinositol (PI)/BMP in a molar ratio of 5/2/1/2; **LEM-Chol composition**, PC/PE/PI/BMP/Chol in a molar ratio of 4/1/1/2/2) and from binary mixtures of PC with either BMP, PG, or PS in a 7/3 molar ratio. To form large unilamellar liposomes, lipid mixtures dissolved in benzene/methanol (95:5) were frozen in liquid nitrogen and then freeze-dried under vacuum overnight. Dry lipid powder was re-suspended in TrN buffer by vigorous vortexing. We subjected the lipid suspension to 10 freeze-thaw cycles by alternating immersion into liquid nitrogen and hot water. Finally, we extruded the lipid suspension 10 times through double-stacked track-etched 100 nm-pore polycarbonate filters (GE Osmonics) using a LIPEX extruder. Liposome sizes were checked using dynamic light scattering on N4 plus submicron particle size analyzer (Beckman Coulter, USA). Liposomes were kept on ice and used within a day.

### Virus binding

In all experiments that involved virus-to-cell binding we incubated cells with viruses at 10°C. We found this temperature to be optimal for DEN binding because at lower temperatures (for instance, at 4°C) viral binding was very inefficient, and at higher temperatures we observed internalization of viral particles by cells.

To compare cell binding for DEN with that for SIN, we surface-biotinylated DEN and SIN particles by incubating viruses (10^6^ IU) with 10 mM EZ-Link Sulfo-NHS-SS-Biotin (Pierce Biotechnology, Rockford, IL) in 0.5 mL phosphate buffer solution (PBS) supplemented with 20 mM Tricine, pH 7.8 (PBS-T buffer) for 30 min at room temperature. The reaction was quenched with 100 mM glycine in PBS. Biotinylated virus (10^5^ IU of DEN or SIN) was allowed to bind to lifted CHO-K1 cells (10^4^ cells) at 10°C for 30 min. Cells were washed three times with cold serum-free ADMEM medium supplemented with 1% BSA by pelleting at 4°C. After removing the unbound virus, we incubated the cells with streptavidin Alexa Fluor 488 conjugate (Molecular Probes, Eugene, OR) as recommended by the manufacturer and carried out flow cytometry analysis using a FACSCanto fluorescence-activated cell sorter (BD Biosciences).

In another experimental approach, we incubated the C6/36 and MA104 cells with DiD-labeled virus in 35 mm plates at 10°C for 1 hr in the ADMEM medium. Unbound viral particles were removed by washing, treated with 2.5 µM NBD-PC at 4°C for 5 min. The cells were washed with cold PBS. Then the cells were lysed and DiD fluorescence dequenched by replacing PBS with 200 µl of PBC supplemented with 1% Triton X100. Samples were cleared from debris by centrifugation. C6/36 cells are much smaller than MA104 cells. To compare surface densities of bound DEN particles at plasma membrane of C6/36 and MA104 cells, we normalized the DiD fluorescence that provides a measure of the amount of bound virus to the NBD fluorescence that provides a measure of the total area of plasma membranes accessible for NBD-PC insertion. This normalization is based on the assumption that NBD-PC similarly partitions into the outer leaflets of plasma membrane bilayers of different cells.

### Viral infection assay

The effects of AL on virus infection were quantified in MA104, Vero and BHK21 cells by fluorescent focus assay. Briefly, serial dilutions of DEN in the ADMEM medium were incubated with confluent monolayers of the cells at 10°C for 60 min. After removing unbound viruses, the cells were treated with 2.5 µM of PS or PG in the ADMEM medium for 5 min at 4°C. The cells were incubated at 37°C for 1 hour in the ADMEM medium, then overlaid with the medium containing 2% FBS and supplemented with 0.75% carboxymethyl-cellulose. The cells were grown for 3 days at 37°C and fluorescent focus units were detected by immunostaining with the primary monoclonal antibody 4G2 and fluorescent secondary antibodies against mouse IgG. Data were normalized to control infection observed for the cells not treated with any exogenous lipids.

### Cell–cell fusion assays

Cells grown to high confluency were incubated with DEN or SIN in complete medium for 30 min at 10°C. Unbound virus was removed by three washes with cold PBS-T, and fusion was triggered at room temperature by application of serum-free ADMEM medium adjusted to different pH values with MES and acetic acid. In unsuccessful attempts to achieve DEN-mediated fusion of mammalian cells, we increased the MOI to 1,000 and extended the duration of the low-pH application to 15 min.

To detect virus-mediated fusion between mammalian cells by redistribution of aqueous contents, we co–plated cells expressing either EGFP or mRedFP at a 1∶1 ratio. A day later, we incubated the cells with virions (DEN, MOI of 300 or SIN, MOI of 40) as described above, applied a 5-min low-pH pulse, and incubated cells for 30 more minutes in the complete medium at 37°C. The average number of co-labeled cells per microscopic field was normalized to the average number of contacts between differently labeled cells in the control experiment, in which cells were not treated with low pH. For each condition, we carried out 3 independent experiments and analyzed at least 10 microscopic fields in each experiment.

To score fusion between mosquito cells C6/36 with bound DEN (MOI of 100) or SIN (MOI of 40) virions we treated the cells with media of different pH for 15 min and then re-neutralized the cells. After a 2-hour incubation at 37°C in complete medium we quantified the efficiency of syncytium formation by measuring a decrease in the number of mononucleated cells. More than 10 fields of view were analyzed for each experimental condition.

In the experiments on virus-mediated HAb2-RBC fusion, cells with associated virions were incubated with PKH26-labeled RBCs to achieve 0–2 bound RBC per cell [62]. After three washes with PBS-T to remove unbound RBC, HAB2–virion–RBC complexes were treated with PBS titrated with citrate to an acidic pH for 5 min and then re-neutralized with PBS-T. Fusion was quantified as the ratio of dye-redistributed bound RBC to the total number of bound RBC.

While the inability of DEN to fuse mammalian cells is illustrated in the figures only for CHO-K1 cells and Vero cells and HAb2-RBC fusion, we carried out similar experiments and observed no DEN-mediated fusion for MA104, BHK21, BS-C-1, RAW 264.7, U967, and NIH3T3 cells.

### Virus–plasma membrane fusion

Fusion of DEN virions to the plasma membrane of MA104 cells, Vero and BHK-21 cells was evaluated by a fusion-infection assay (FIA) [34]. This assay is based on measuring infection caused by low-pH-induced fusion between viral particles and plasma membrane under conditions when endocytotic entry of virus is blocked by inhibitors of endosomal acidification. We plated the cells on Lab-tek II 8-well chambered coverglass (Nalge Nunc) a day before experiment, then pre-treated them with 50 µM chloroquine (Sigma, St. Louis, MO) for 30 min at 37°C. Viral particles were allowed to bind to the cell surfaces at 10°C for 60 min (MOI  =  300). After removal of unbound virus, the cells were treated (or not treated) with 2.5 µM of PS or PG in the ADMEM medium for 5 min at 4°C. Fusion was triggered at room temperature by replacing the medium with the ADMEM medium acidified to pH 5.3. 5 min later the cells were neutralized, incubated at 37°C for 4 hours in the ADMEM medium supplemented with chloroquine. By that time, virions that did not infect the cells by low pH-induced fusion to plasma membrane, have been internalized and, because of the blocked endosomal acidification and, thus, fusion, have already passed the endosomal compartments allowing productive RNA release and infection. After this 4 hour incubation in the presence of chloroquine, we overlaid the cells with the ADMEM medium supplemented with 2% FBS and 0.75% carboxymethyl cellulose to prevent virus spread. Fluorescent focus units were detected 3 days later by immunostaining with the antibody 4G2. Data were normalized to those obtained for the cells that were not treated with AL. We found FIA assay for DEN and mammalian cells to be very sensitive to the concentration of chloroquine (and other inhibitors of endosomal acidification) and the duration of the application of these inhibitors (longer applications results in cytotoxity) and to MOI used.

### Intracellular fusion assay

MA104 and BS-C-1 cells were grown on the coverglass bottom of 35-mm tissue culture dishes (MatTek, MA) to high confluency. DiD-labeled virus was added to cells (MOI of 100) and allowed to bind for 30 min at 10°C. Unbound virus was removed by three washes with cold PBS-T. The cells were warmed up to 37°C to allow virus internalization. After incubation of cells at 37°C for different times, the cells were fixed with 4% paraformaldehyde and analyzed by fluorescence microscopy. Fusion of DiD labeled virus within endosomes leads to dequenching of DiD and appearance of bright fluorescent spots throughout the whole cell but mostly in the perinuclear region. To quantify fusion efficiency, we imaged cells with an iXon^EM^+ 885 EMCCD Camera (Andor Technology, CT) on an AxiObserver inverted fluorescence microscope (Zeiss, Germany) using a Cy5-4040A filter set (Semrock, NY). To capture signal from all fused viruses, we collected image stacks throughout the cell with 250 nm spacing between slices. We analyzed the acquired images using an ImageJ macro developed in-house to measure the total fluorescence signal from bright spots per imaging field (20 fields per experimental condition were analyzed in each independent experiment; each experiment was repeated at least three times).

### Virus-liposome fusion

We assayed lipid mixing between DiD labeled viral particles and liposomes as DiD dequenching in four-clear-sided methacrylate cuvettes (Fisher Scientific, Pittsburgh, PA). The medium in the cuvettes was continuously stirred with a magnetic stirring device and thermostatted at 37°C. We mixed 10 µl (∼10^5^ IU) of purified labeled virus with liposomes (final concentration of lipid 30 µM) in 2 ml of TrN buffer. We initiated the fusion reaction by adding a pre-titrated amount of MES/acetic acid buffer to reach the desired pH. We recorded fluorescence for at least 15 min at excitation and emission wavelengths of 620 and 665 nm, respectively, using an Aminco Bowman Series 2 luminescence spectrometer (Rochester, NY). At the end of each recording, we added Triton X-100 to a final concentration of 0.1% to fully dequench DiD (“100% lipid mixing”). We routinely verified that under our conditions the efficiency of lipid mixing (rates and extents) was not limited by the concentration of the liposomes used.

### Virus inactivation by DEPC

DEN was inactivated by a 15-min incubation at room temperature in PBS-T supplemented with 2 mM DEPC (Sigma, St. Louis, MO) added from freshly prepared stock solution in cold ethanol. DEPC was reported to inhibit vesicular stomatatis virus by modifying histidine residues on viral protein fusogen [25].

### Exogenous lipids

To add exogenous lipids (PS or PC or PG) to plasma membranes of CHO-K1, MA104, BS-C-1 cells and HAb2–RBC pairs, we incubated the cells with associated virions in a PBS supplemented with 16:0-06:0 NBD PS (1-palmitoyl-2-{6-[(7-nitro-2-1,3-benzoxadiazol-4-yl)amino]hexanoyl}-*sn*-glycero-3-phosphoserine), 16:0-06:0 NBD PC (1-palmitoyl-2-{6-[(7-nitro-2-1,3-benzoxadiazol-4-yl)amino]hexanoyl}-*sn*-glycero-3-phosphocholine) or 16:0-06:0 NBD PG (1-palmitoyl-2-{6-[(7-nitro-2-1,3-benzoxadiazol-4-yl)amino]hexanoyl}-*sn*-glycero-3-[phospho-*rac*-(1-glycerol)]), all lipids purchased from Avanti Polar Lipids, Alabaster, AL). A 2.5 mM stock solution (1 µl) of PS, PC or PG in ethanol was injected into 1 ml of PBS-T under intensive vortexing. The cells were cooled down to 4°C and placed into this lipid-supplemented medium for 5 min, still at 4°C. We inferred from the levels of cell-associated NBD fluorescence observed with fluorescence microscopy that PS, PC and PG incorporated into cell membranes to similar concentrations (see also [64]). For virus-mediated cell–cell fusion, the lipid-supplemented buffer was replaced with warm (room temperature) serum-free ADMEM medium adjusted to different pH values by titration with MES and acetic acid. After the end of low pH application the cells were incubated at 37°C for 30 min and fusion was quantified by fluorescence microscopy. We verified that at the time of low pH application a significant part of exogenous lipids remain extractable by delipidated BSA. For the intracellular fusion assay, the cells with bound DiD-labeled DEN or SIN virions were treated or not treated with exogenous lipids as described above. The temperature was raised and, after incubation at 37°C for different times, we fixed the cells and analyzed them by fluorescence microscopy.

### CPZ

To reveal DEN-mediated RH between HAb2 cells and PKH26-labeled RBCs, a 5-min low pH pulse was followed by a 1-min application of a 0.5 mM solution of CPZ (Sigma, St. Louis, MO) in PBS-T. The percentage of HAb2–RBC pairs demonstrating lipid mixing (PKH redistribution from RBC to HAb2 cell) was assayed with fluorescence microscopy 20 min after the end of the low–pH pulse.

### Annexin V

Annexin V is widely used to evaluate the expression of PS on cell surfaces [65]. We used this protein in two different experimental approaches. To inhibit DEN interactions with PS in the outer leaflet of plasma membranes of C6/36 cells, we first incubated the cells in annexin-binding buffer (BD Pharmingen, San Jose, CA) at room temperature for 30 min and then allowed DEN or SIN virions to bind to the cells. After removal of unbound virions, we incubated the cells with 50 µg/ml recombinant annexin V (BD Pharmingen, San Jose, CA) for 30 min at 10°C, washed the cells from unbound annexin, and then triggered fusion by applying an acidic pH medium. We verified that annexin V treatment had no effect on virus–cell binding using DiD-labeled virions.

To compare PS expression at the surfaces of different cells, we used R-phycoerythrin -tagged annexin V (BD Pharmingen, San Jose, CA), as recommended by the manufacturer.

### Lysosomotropic agents

In some experiments on intracellular fusion, to block endosomal acidification we treated the cells with the lysosomotropic agents chloroquine (Sigma, St. Louis, MO; 50 µM, 30 min, 37°C) or bafilomycin-A1 (Sigma, St. Louis, MO; 2 µM, 30 min, 37°C) prior to applying viral inoculum.

### Dominant-negative Rab7

Cells were transfected using Lipofectamine 2000 (Invitrogen) according to the manufacturer's instructions with a EGFP-Rab7a S22N plasmid [66], a kind gift from Julie Donaldson, NIH, on Lab-tek II 4-well chambered coverglass (Nalge Nunc). 18 hours later, DiD-labeled viral particles were allowed to bind to the cell surfaces at 10°C for 60 min. After removal of unbound virus, cells were treated (or not treated) with 2.5 µM of PS in the ADMEM medium for 5 min at 4°C and then incubated in the ADMEM medium at 37°C for 40 min. Cells were fixed and nuclei were stained with DAPI (Invitrogen). Analysis by fluorescence microscopy allowed us to identify the transfected cells by their EGFP fluorescence and to detect DEN fusion within endosomal pathway as intracellular structures displaying DiD fluorescence.

### Nocodazole

MA104 cells were preincubated with nocodazole (Sigma, St. Louis, MO; 60 µM in complete medium, 30 min, 37°C) prior to application of DEN and exogenous lipids. Nocodazole was present throughout the entire experiment.

## Supporting Information

Figure S1 DEN binding to C6/36 cells and MA104 cells. After incubation of DiD-labeled virus with the cells at 10°C, the cells were washed to remove unbound virions, the temperature was lowered to 4°C and NBD-PC was applied. After washing the cells, we lysed them and measured DiD- and NBD- fluorescences. To compare surface densities of bound DEN particles at plasma membrane of the cells of the different sizes (C6/36 cells are much smaller than MA104 cells), we normalized the DiD fluorescence that provides a measure of the amount of bound virus to the NBD fluorescence that provides a measure of the total area of plasma membranes accessible for NBD-PC insertion. The data for C6/36 are taken as 100%. The data are presented as means +/- s.d, n = 3.
(0.03 MB PDF)

Figure S2 The dependence of DEN-4 fusion on liposome composition. Lipid mixing between DiD-labeled DEN-4 virions and liposomes of LEM and PM compositions (pH 5.6, 37°) assayed as DiD dequenching. In the negative controls, we measured lipid mixing for LEM liposomes and DEN-4 virions inactivated either by pre-incubation at pH 4.5 or by DEPC.
(0.07 MB PDF)

Figure S3 The dependence of SIN fusion to liposomes on anionic lipids. Low-pH-dependent lipid mixing between DiD-labeled SIN particles and liposomes of LEM-Chol (green bars) or PM (red bars) compositions was measured at 37°C as a dequenching of DiD fluorescence at pH 5.3. No lipid mixing was observed at neutral pH (striped bands). The data are presented as extents of lipid mixing 10 min after acidification and the initial rates of the lipid mixing (means +/− s.d, n = 3).
(0.04 MB PDF)

Figure S4 The dependence of SIN-mediated fusion of CHO-K1 cells on anionic lipids. CHO-K1 cells carrying SIN virions at their surface were treated with PS or PG or not treated with exogenous lipids (PS, PG, Low pH) immediately prior to a 5-min application of pH 5.3. No fusion was observed if the cells were not exposed to low pH (No low pH). After 30 min incubation in the complete medium at 37°C, fusion was assayed with fluorescence microscopy as the appearance of co-labeled cells. The average number of fusion events ( =  the number of co-labeled cells) per microscopic field was normalized to the average number of contacts between differently labeled cells per field in the control experiment, in which cells were not treated with low pH. For each condition, we analyzed at least 10 microscopic fields.
(0.03 MB PDF)

Figure S5 Cell-surface labeling with Alexa Fluor 488-conjugated annexin V for C6/36 cells than for Vero cells. The cells were pre-incubated with annexin-binding buffer for 30 min at room temperature and fluorescent annexin was applied in the same buffer at concentration recommended by Invitrogen. Two panels were photographed under the same settings to allow direct comparison.
(0.88 MB PDF)

Figure S6 An increase in the rate of DEN fusion within endocytic pathway for cells treated with exogenous PS. BS-C-1 cells were incubated with DiD-labeled DEN at 10°C for 30 min to allow binding but not internalization of the virions. Unbound virus was removed and the temperature was raised to 37°C to allow virus to enter cells. Viral fusion events along the endocytic pathway resulted in DiD dequenching and were detected as appearance of fluorescent spots within the cells. **A.** - DiD fluorescence (top panel) and phase contrast (bottom panel) images of the cells treated and untreated with 2.5 µM of 16:0-06:0 NBD PS (right and left images, respectively) taken 30 min after raising the temperature. Scale bar, 25 µm. **B.** The cells were fixed at different times after raising the temperature. Curves present time course of an increase in the mean total fluorescence of the bright spots per imaging field for untreated BS-C-1 cells (curve 2) and for cells treated with 2.5 µM of 16:0-06:0 NBD PS (curve 1). Each point is based on analysis of 20 fields for each experimental condition in each of 3 independent experiments. The data are normalized to the mean fluorescence observed for PS-treated cells 30 min after raising the temperature and presented as mean +/− s.d.
(0.38 MB PDF)

Figure S7 Inhibition of intracellular fusion of DiD-labeled DEN in MA104 cells transfected with dominant negative EGFP-tagged Rab7a S22N is alleviated by treating the cells with PS. **A.** In contrast to surrounding cells, identified by the presence of DAPI-stained (blue) nuclei, DN Rab7a-expressing cell, identified by its green (EGFP) fluorescence, contains almost no DiD labeled (orange) structures. **B.** Image on the right. Treating the cells with PS alleviated the DN Rab7a inhibition of DEN fusion, as evidenced by multiple DiD labeled structures observed within a green (DN Rab7a-expressing) PS-treated cell. Image on the left, taken with the same settings as image on the right, shows, similarly to **A**, the lack of DiD structures in DN Rab7a expressing cell.
(0.29 MB PDF)

Figure S8 In SIN-mediated cell fusion, transition from the restricted hemifusion to lipid mixing at optimal pH requires neither PS nor CPZ application. pH dependence of the SIN-mediated fusion for the cells treated or not treated with PS and/or with CPZ (0.5 mM, applied for 1 min). HAb2-SIN-PKH26-labeled RBC complexes were treated for 5 min with medium of a given pH to trigger conformational changes in protein fusogens and, thus, fusion. Fusion was assayed as a percentage of HAb2-SIN-RBC complexes demonstrating lipid mixing 20 min after the end of the low pH application. (1) - HAb2-SIN-RBC complexes treated with neither PS nor CPZ. (2) - HAb2-SIN-RBC complexes treated with PS immediately prior to low pH application. (3) - HAb2-SIN-RBC complexes treated with CPZ pulse immediately after the end of low pH application. (4) - HAb2-SIN-RBC complexes treated with PS immediately prior to low pH application and with CPZ pulse immediately after the end of low pH application. The data presented as mean +/− s.d., n = 3.
(0.04 MB PDF)
