# Supplementary figures and images for: Dengue Virus Ensures Its Fusion in Late Endosomes Using Compartment-Specific Lipids
Source: PLoS Pathog. 2010 Oct 7;6(10):e1001131. doi: 10.1371/journal.ppat.1001131 (PMC2951369; doi:10.1371/journal.ppat.1001131)

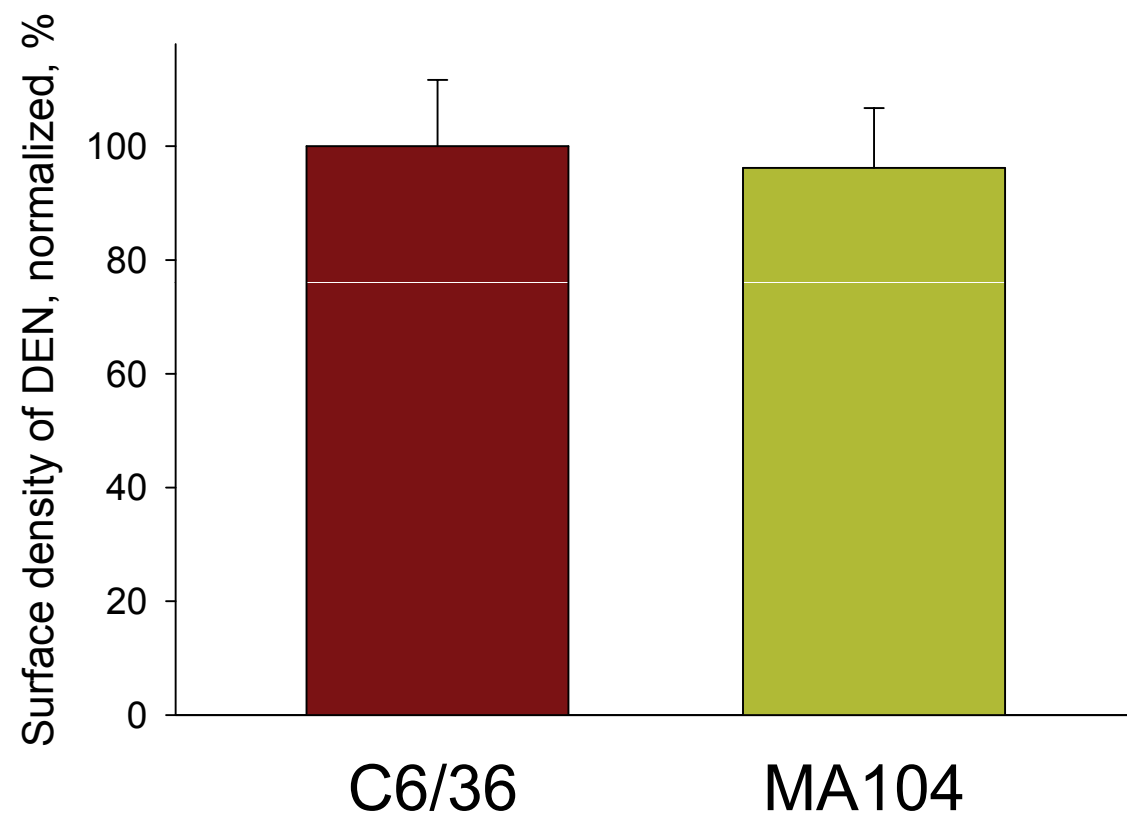

Supplement: Figure S1 — DEN binding to C6/36 cells and MA104 cells. After incubation of DiD-labeled virus with the cells at 10°C, the cells were washed to remove unbound virions, the temperature was lowered to 4°C and NBD-PC was applied. After washing the cells, we lysed them and measured DiD- and NBD- fluorescences. To compare surface densities of bound DEN particles at plasma membrane of the cells of the different sizes (C6/36 cells are much smaller than MA104 cells), we normalized the DiD fluorescence that provides a measure of the amount of bound virus to the NBD fluorescence that provides a measure of the total area of plasma membranes accessible for NBD-PC insertion. The data for C6/36 are taken as 100%. The data are presented as means +/- s.d, n = 3. (0.03 MB PDF) [file ppat.1001131.s001.pdf]

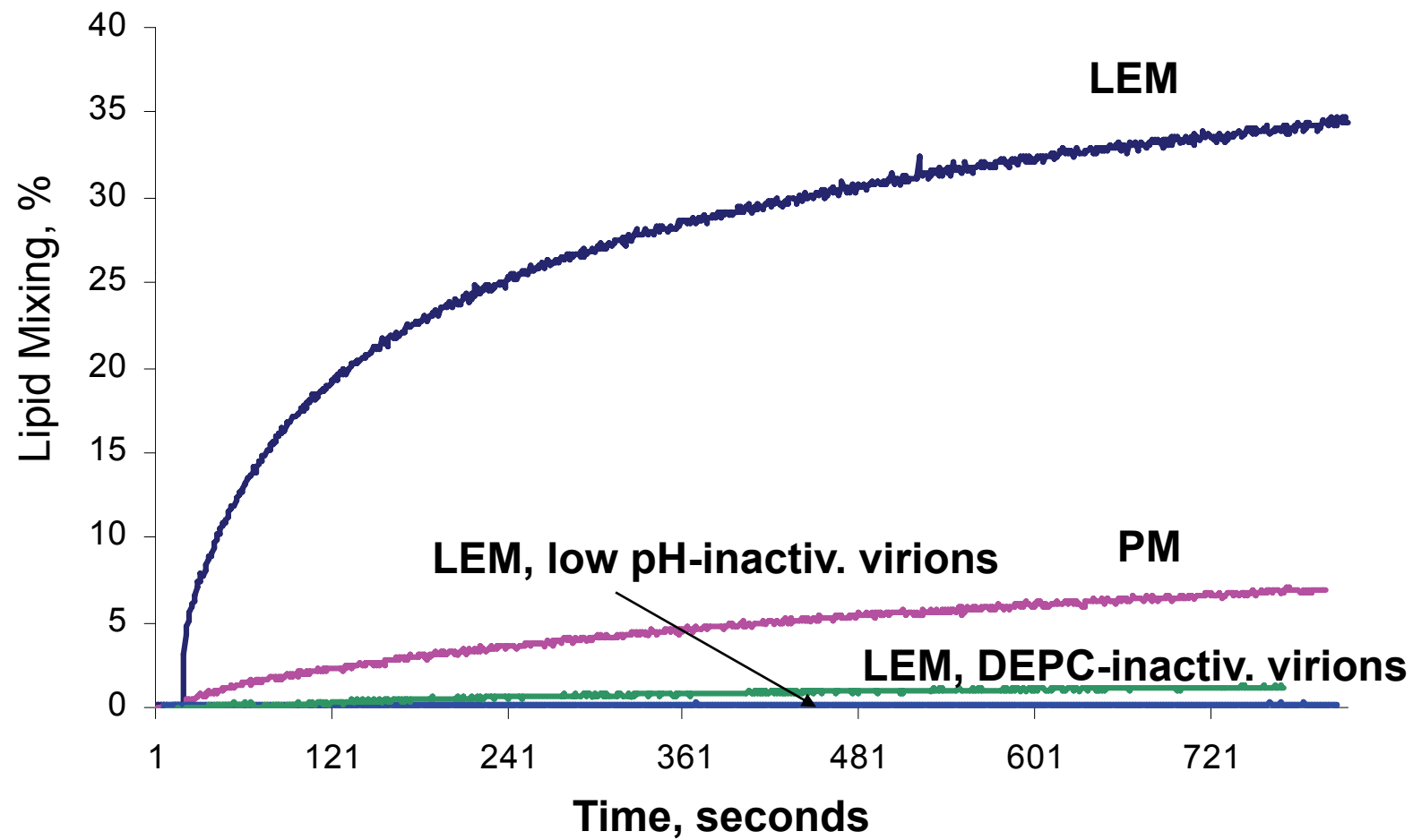

Supplement: Figure S2 — The dependence of DEN-4 fusion on liposome composition. Lipid mixing between DiD-labeled DEN-4 virions and liposomes of LEM and PM compositions (pH 5.6, 37°) assayed as DiD dequenching. In the negative controls, we measured lipid mixing for LEM liposomes and DEN-4 virions inactivated either by pre-incubation at pH 4.5 or by DEPC. (0.07 MB PDF) [file ppat.1001131.s002.pdf]

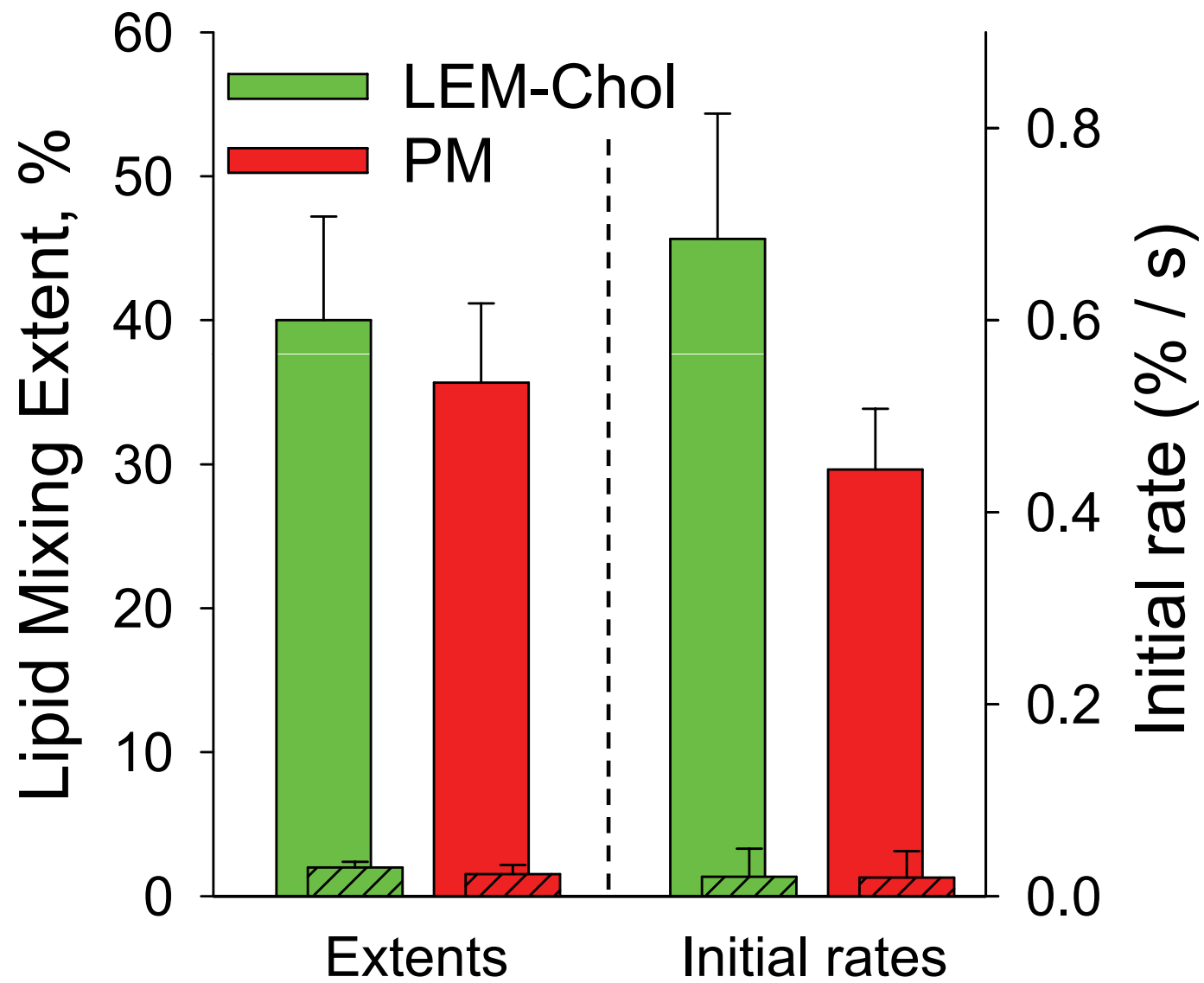

Supplement: Figure S3 — The dependence of SIN fusion to liposomes on anionic lipids. Low-pH-dependent lipid mixing between DiD-labeled SIN particles and liposomes of LEM-Chol (green bars) or PM (red bars) compositions was measured at 37°C as a dequenching of DiD fluorescence at pH 5.3. No lipid mixing was observed at neutral pH (striped bands). The data are presented as extents of lipid mixing 10 min after acidification and the initial rates of the lipid mixing (means +/− s.d, n = 3). (0.04 MB PDF) [file ppat.1001131.s003.pdf]

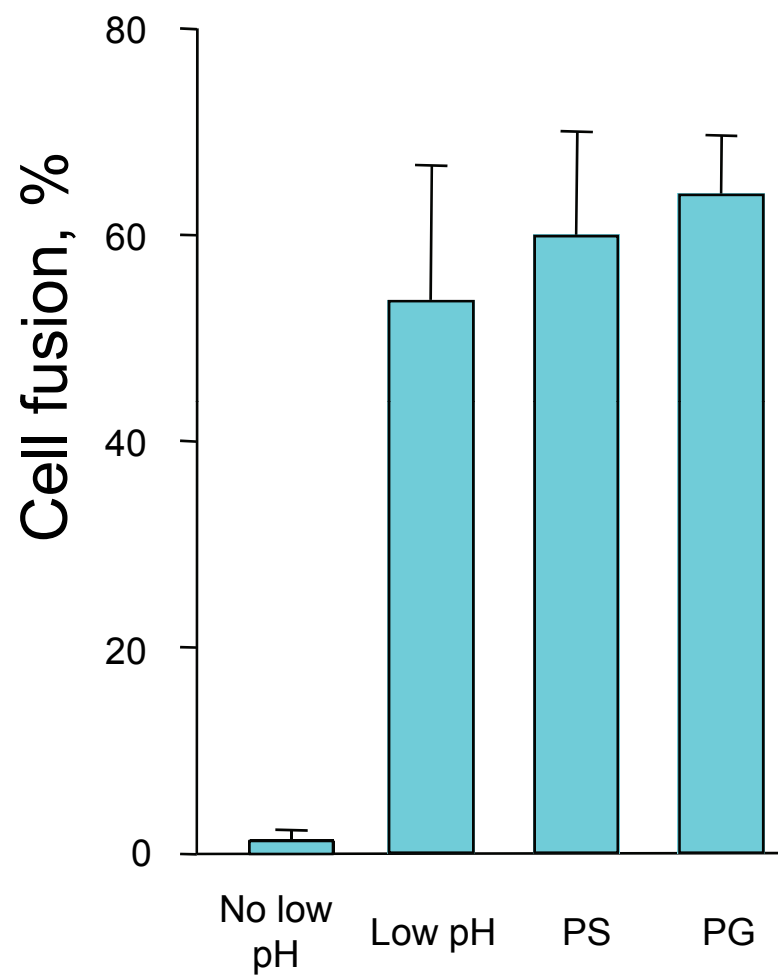

Supplement: Figure S4 — The dependence of SIN-mediated fusion of CHO-K1 cells on anionic lipids. CHO-K1 cells carrying SIN virions at their surface were treated with PS or PG or not treated with exogenous lipids (PS, PG, Low pH) immediately prior to a 5-min application of pH 5.3. No fusion was observed if the cells were not exposed to low pH (No low pH). After 30 min incubation in the complete medium at 37°C, fusion was assayed with fluorescence microscopy as the appearance of co-labeled cells. The average number of fusion events ( = the number of co-labeled cells) per microscopic field was normalized to the average number of contacts between differently labeled cells per field in the control experiment, in which cells were not treated with low pH. For each condition, we analyzed at least 10 microscopic fields. (0.03 MB PDF) [file ppat.1001131.s004.pdf]

C6/36

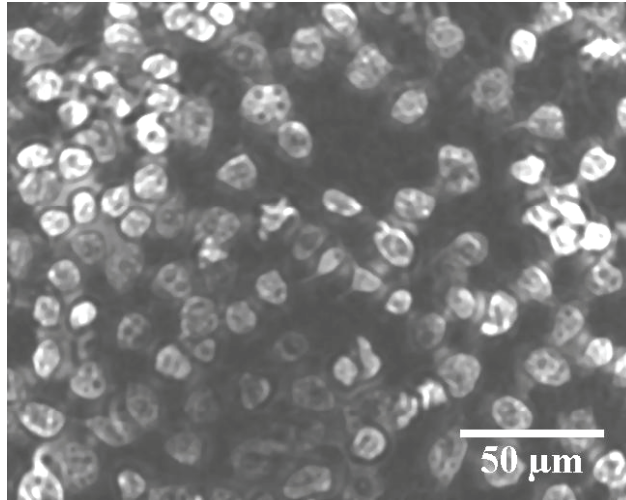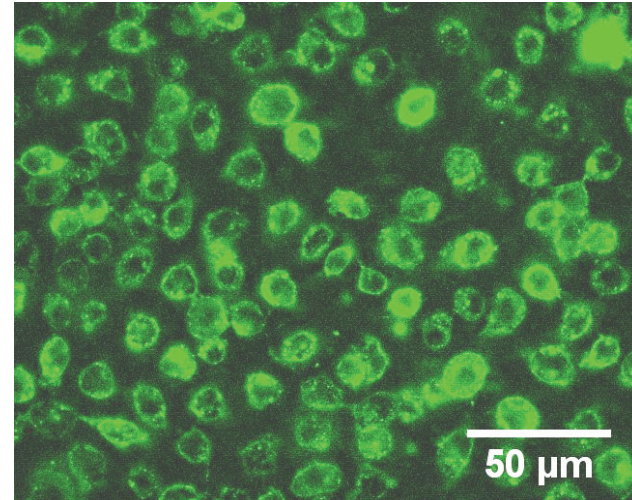

Vero

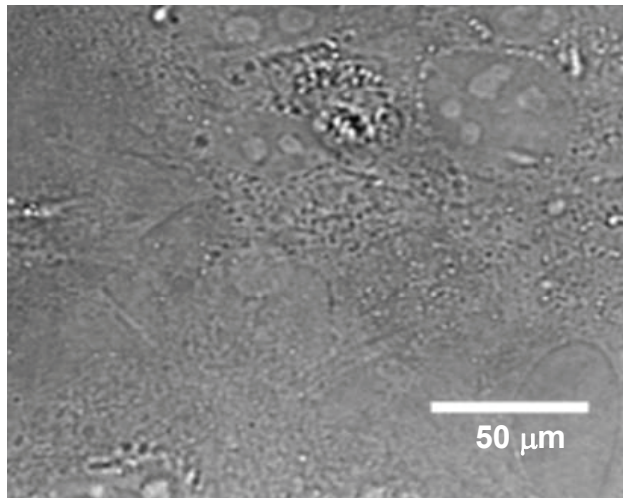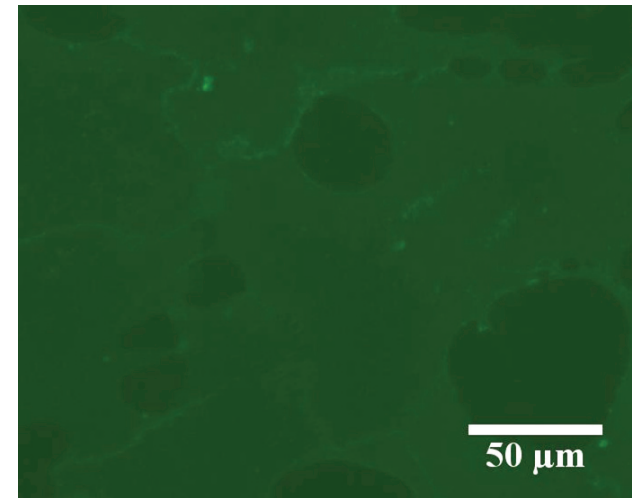

Supplement: Figure S5 — Cell-surface labeling with Alexa Fluor 488-conjugated annexin V for C6/36 cells than for Vero cells. The cells were pre-incubated with annexin-binding buffer for 30 min at room temperature and fluorescent annexin was applied in the same buffer at concentration recommended by Invitrogen. Two panels were photographed under the same settings to allow direct comparison. (0.88 MB PDF) [file ppat.1001131.s005.pdf]

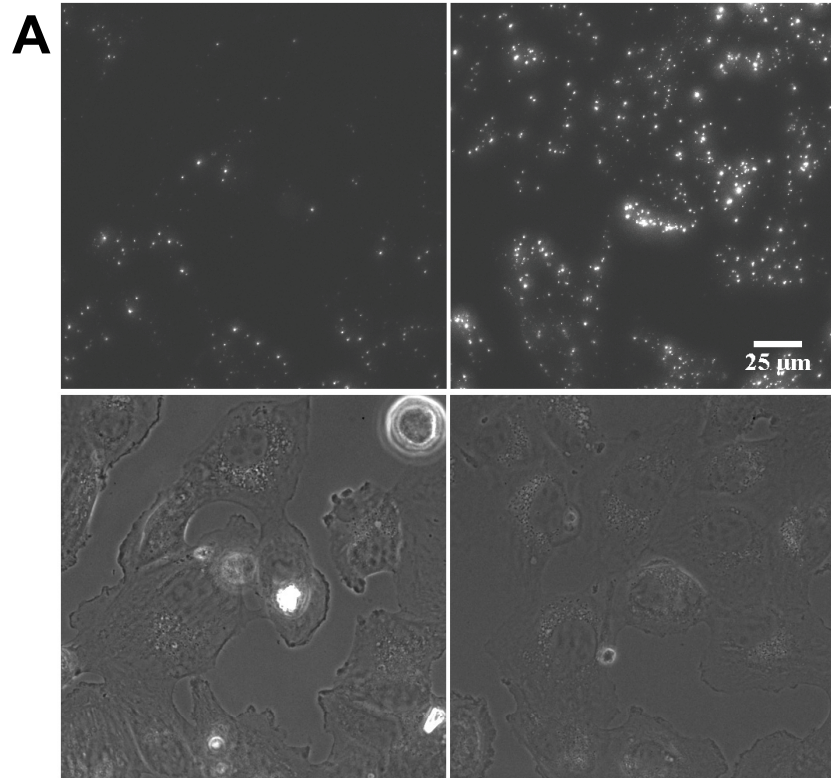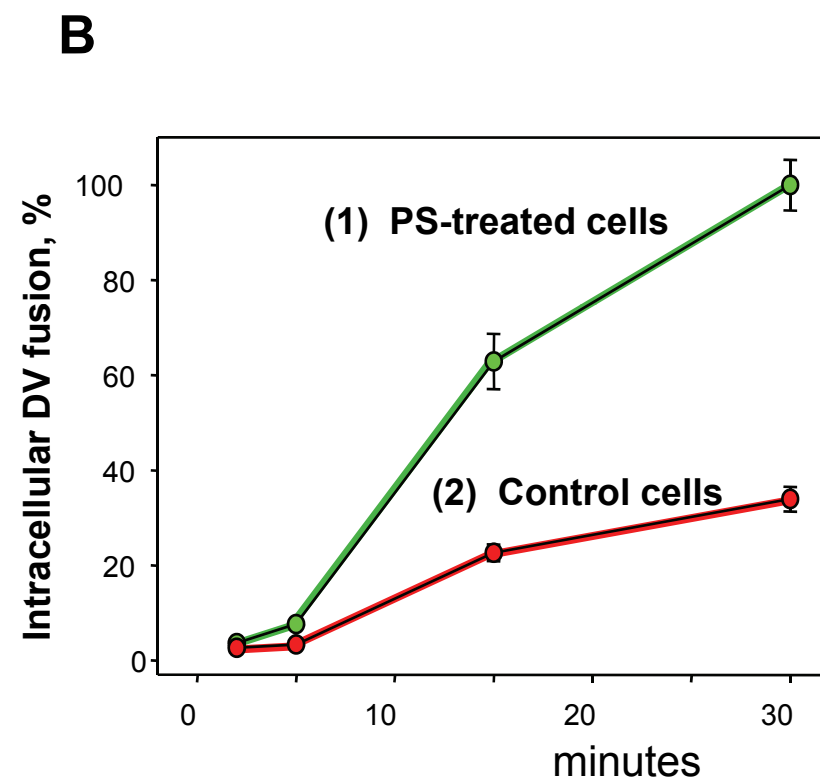

Supplement: Figure S6 — An increase in the rate of DEN fusion within endocytic pathway for cells treated with exogenous PS. BS-C-1 cells were incubated with DiD-labeled DEN at 10°C for 30 min to allow binding but not internalization of the virions. Unbound virus was removed and the temperature was raised to 37°C to allow virus to enter cells. Viral fusion events along the endocytic pathway resulted in DiD dequenching and were detected as appearance of fluorescent spots within the cells. A. - DiD fluorescence (top panel) and phase contrast (bottom panel) images of the cells treated and untreated with 2.5 µM of 16:0-06:0 NBD PS (right and left images, respectively) taken 30 min after raising the temperature. Scale bar, 25 µm. B. The cells were fixed at different times after raising the temperature. Curves present time course of an increase in the mean total fluorescence of the bright spots per imaging field for untreated BS-C-1 cells (curve 2) and for cells treated with 2.5 µM of 16:0-06:0 NBD PS (curve 1). Each point is based on analysis of 20 fields for each experimental condition in each of 3 independent experiments. The data are normalized to the mean fluorescence observed for PS-treated cells 30 min after raising the temperature and presented as mean +/− s.d. (0.38 MB PDF) [file ppat.1001131.s006.pdf]

**A**

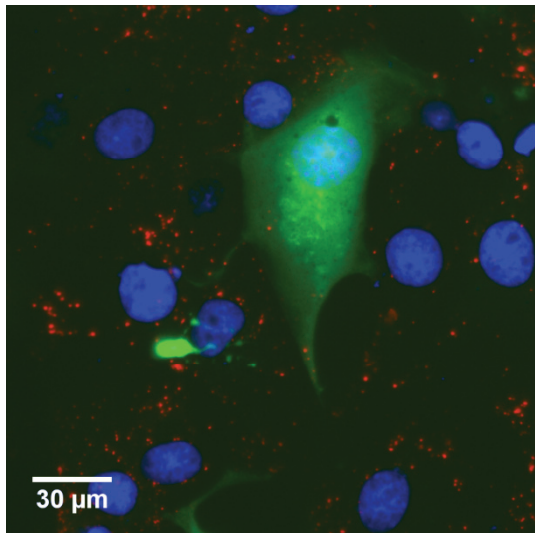

**B**

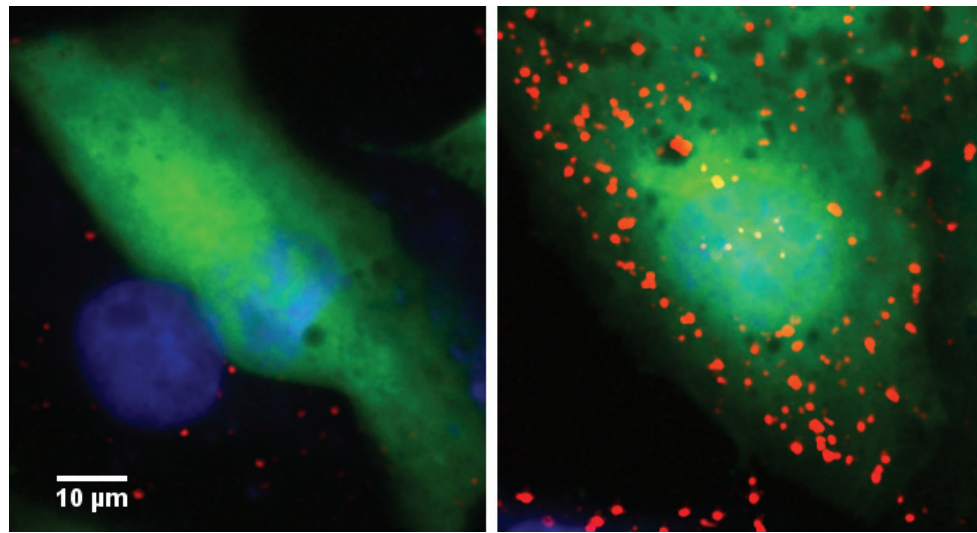

Supplement: Figure S7 — Inhibition of intracellular fusion of DiD-labeled DEN in MA104 cells transfected with dominant negative EGFP-tagged Rab7a S22N is alleviated by treating the cells with PS. A. In contrast to surrounding cells, identified by the presence of DAPI-stained (blue) nuclei, DN Rab7a-expressing cell, identified by its green (EGFP) fluorescence, contains almost no DiD labeled (orange) structures. B. Image on the right. Treating the cells with PS alleviated the DN Rab7a inhibition of DEN fusion, as evidenced by multiple DiD labeled structures observed within a green (DN Rab7a-expressing) PS-treated cell. Image on the left, taken with the same settings as image on the right, shows, similarly to A, the lack of DiD structures in DN Rab7a expressing cell. (0.29 MB PDF) [file ppat.1001131.s007.pdf]

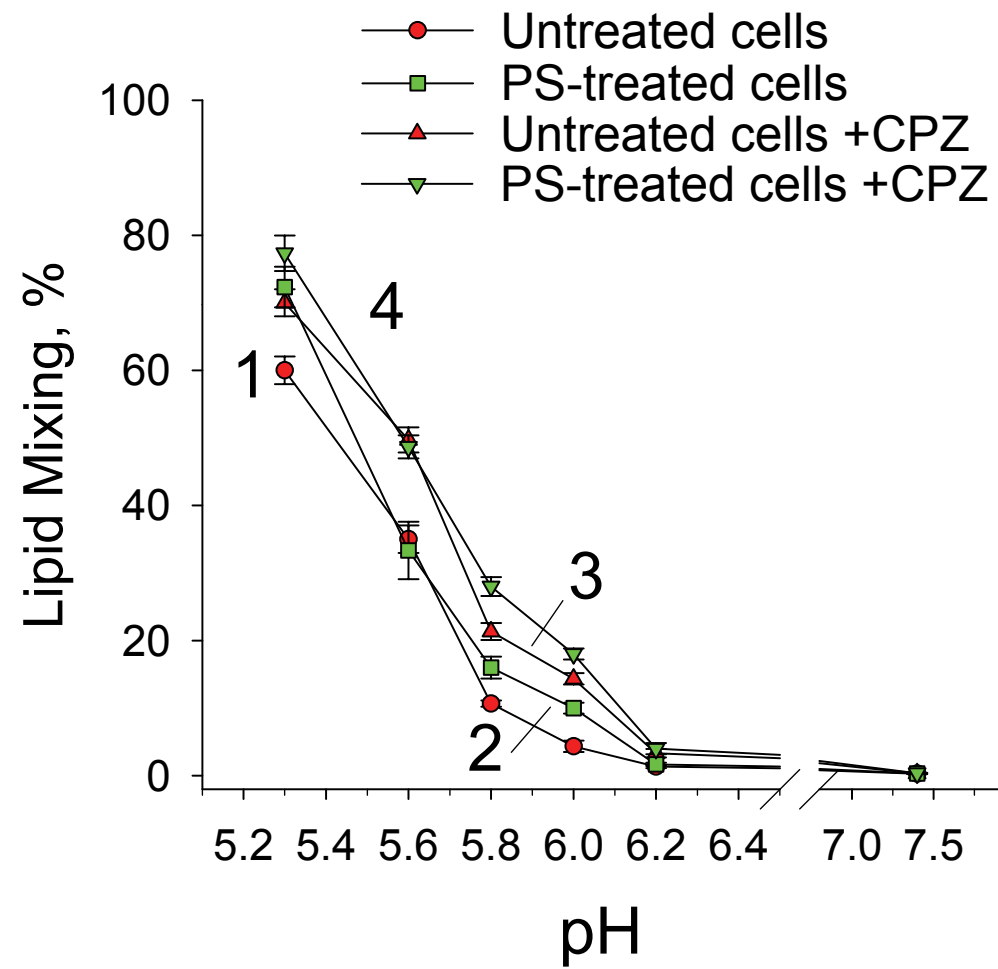

Supplement: Figure S8 — In SIN-mediated cell fusion, transition from the restricted hemifusion to lipid mixing at optimal pH requires neither PS nor CPZ application. pH dependence of the SIN-mediated fusion for the cells treated or not treated with PS and/or with CPZ (0.5 mM, applied for 1 min). HAb2-SIN-PKH26-labeled RBC complexes were treated for 5 min with medium of a given pH to trigger conformational changes in protein fusogens and, thus, fusion. Fusion was assayed as a percentage of HAb2-SIN-RBC complexes demonstrating lipid mixing 20 min after the end of the low pH application. (1) - HAb2-SIN-RBC complexes treated with neither PS nor CPZ. (2) - HAb2-SIN-RBC complexes treated with PS immediately prior to low pH application. (3) - HAb2-SIN-RBC complexes treated with CPZ pulse immediately after the end of low pH application. (4) - HAb2-SIN-RBC complexes treated with PS immediately prior to low pH application and with CPZ pulse immediately after the end of low pH application. The data presented as mean +/− s.d., n = 3. (0.04 MB PDF) [file ppat.1001131.s008.pdf]
